# Methodological Quality Assessment of Budget Impact Analyses for Orphan Drugs: A Systematic Review

**DOI:** 10.3389/fphar.2021.630949

**Published:** 2021-04-21

**Authors:** Khadidja Abdallah, Isabelle Huys, Kathleen Claes, Steven Simoens

**Affiliations:** ^1^ Department of Pharmaceutical and Pharmacological Sciences, KU Leuven, Leuven, Belgium; ^2^ Department of Microbiology, Immunology and Transplantation, UZ Leuven, Leuven, Belgium

**Keywords:** budget impact analyses, orphan drugs, guidelines, quality assesment, systematic reviews

## Abstract

**Objectives:** This research aims to evaluate the methodological quality of budget impact analyses for orphan drugs and to provide suggestions for future analyses.

**Methods:** Conference abstracts and peer-reviewed literature on budget impact analyses were collected through searches of Pubmed and Embase. ISPOR good practice guidelines were used as a methodological standard for budget impact analyses. Examined parameters encompassed: perspective, target population, data sources, intervention and comparator(s), time horizon, scope of costs, discounting, validation, assumptions and sensitivity analysis.

**Results:** Seventy studies on individual orphan drugs and 21 studies on a combination of orphan drugs analyzing budget impact were identified. Overall, analyses considered a third-party payer perspective, reported periodic budget impacts over a one-to-five-year time horizon, and did not apply discounting. A dynamically fluctuating population and costs beyond drug costs were accounted for in 18.7% and 51.7% of studies, respectively. Input data were retrieved from published literature, clinical trials, registries, claims databases, expert opinions, historical data and market research. Assumptions were mostly made about population size and intervention/comparator(s) market uptake, but these assumptions were rarely justified and their impact was insufficiently explored through sensitivity analyses. Budget impact results were rarely validated.

**Conclusion:** Existing budget impact analyses for orphan drugs are concise, vary greatly and are of substandard methodological quality. To eliminate possible bias in future budget impact analyses, future studies should adhere to national or ISPOR good practice guidelines on budget impact analysis.

## Introduction

With health care expenditure trends surpassing economic growth rates globally (OECD, 2019; WHO, 2019), concerns about financial sustainability have surged. The third major contributor to total health expenditure worldwide is pharmaceutical spending (OECD, 2019), which augmented from US$ 390.2 billion in 2001 to US$ 1.2 trillion in 2018 and is expected to reach US$ 1.52 trillion by 2023 (IQVIA, 2020a; IQVIA, 2020b). Scientific innovation and technological advances in research have been conducive to pharmaceutical growth (OECD, 2019). Furthermore, the late 20th century was accentuated by incentivization of drug development for rare diseases (FDA, 1983; Official Journal of the European Communities, 2000); in 1999, the European Commission (EC) adopted the Orphan Regulation for drugs used in the prevention, diagnosis and treatment of chronically debilitating and life-threatening diseases with low prevalence of five in 10,000 (Official Journal of the European Communities, 2000). As a result, orphan drug share of European drug market increased from 3.1% to 7.2% between 2010 and 2017. Moreover, orphan drug expenditure grew 16% compared to only 3% growth in total pharmaceutical expenditure from 2001 to 2017 (Mestre-Ferrandiz et al., 2019). This rise in orphan drug expenditure instigated funding challenges revealing inequity in access to orphan drugs amongst lower-income and higher-income EU countries (Szegedi et al., 2018). Hence, orphan drug reimbursement extrema of 29.4% and 92.8% were captured in, respectively, Poland and France (Szegedi et al., 2018). Additionally, public pharmaceutical expenditure on orphan drugs ranged from 2.25% to 6.51% in, respectively, the Czech Republic and Belgium (Szegedi et al., 2018). It has been of political priority for many countries to address the ascending pressure on health care budgets in order to maintain market access of qualitative interventions within their fiscal constraints.

Prior to a health intervention being publicly available, it is subject to a country-specific reimbursement process that relies on rigorous health technology assessment (HTA) (Drummond et al., 2007; Denis et al., 2010b; Nicod et al., 2019). One pillar in this assessment is budget impact analysis (BIA), a method that measures affordability of a health intervention in a specific healthcare system (Sullivan et al., 2014). To obtain a comprehensive profile of the intervention, BIA is recommended conjointly with cost-effectiveness analysis (CEA), which estimates the value of an intervention opposed to an alternative (Garattini and van de Vooren, 2011; Sullivan et al., 2014; Mauskopf and Earnshaw, 2017; Pearson, 2018). This is crucial for interventions such as orphan drugs that otherwise fail cost-effectiveness criteria because of their expensive nature and limited evidence-based performance (Drummond et al., 2007; Nicod et al., 2019). In contrast, annual health expenditures of orphan drugs in Europe are generally low (Schlander et al., 2018), with a maximum per capita spending of €20.23 in France (Hutchings et al., 2014), and are forecasted to stabilize below the market growth rate at 4–5% of total pharmaceutical expenditures (Schey et al., 2011). However, a recent publication could not refute a possible slignificant impact of orphan drugs on future pharmaceutical budgets (Gombocz and Vogler, 2020).

In this light, BIA has been regarded as an important measure of affordability and has increasingly been integrated in reimbursement assessments worldwide (Foroutan et al., 2018). However, this realization was preceded by debates on BIA value within HTA, making it a rather new analysis that is in continuous development (Niezen et al., 2009; Foroutan et al., 2020). Some studies point out the inaccuracy of BIA with often an overestimation of budget impacts (Geenen et al., 2020a; Geenen et al., 2020b). To aid in framing structural elements of BIA, several studies, as well as the International Society for Pharmacoeconomics and Outcomes Research (ISPOR) in 2007 and 2014, released good practice guidelines on BIA (Trueman et al., 2001; Mauskopf et al., 2007; Garattini and van de Vooren, 2011; Sullivan et al., 2014; Silva et al., 2017).

Likewise, several jurisdictions have specific requirements for BIA often adapted from the ISPOR guidelines and interpreted to fit their principles (Marshall et al., 2008; Ferreira-Da-Silva et al., 2012; Neyt et al., 2015; Mauskopf and Earnshaw, 2016; Foroutan et al., 2018; Ghabri et al., 2018; Ghabri and Mauskopf, 2018; HIQA, 2018; Foroutan et al., 2019). In spite of these standardizing efforts, BIA are often found to be simplistic, incomplete, inconsistent and poorly designed (Mauskopf et al., 2005; Orlewska and Gulacsi, 2009; van de Vooren et al., 2014; Faleiros et al., 2016). This inaccurate and non-transparent portrayal of BIA complicates inter- and intranational BIA comparison but could especially lead to misinformed resource allocation while potentially disadvantaging the availability of unconventional treatments like orphan drugs (Orlewska and Mierzejewski, 2004; Marshall et al., 2008; Ferreira-Da-Silva et al., 2012; Neyt et al., 2015; Ghabri et al., 2018; Ghabri and Mauskopf, 2018; HIQA, 2018; Foroutan et al., 2019).

In this study, we systematically identify BIA of orphan drugs in the literature with the aim of assessing the methodological quality of the reported analyses. Based on this review, recommendations are proposed to improve reporting of BIAs for orphan drugs.

## Methods

**FIGURE 1:**
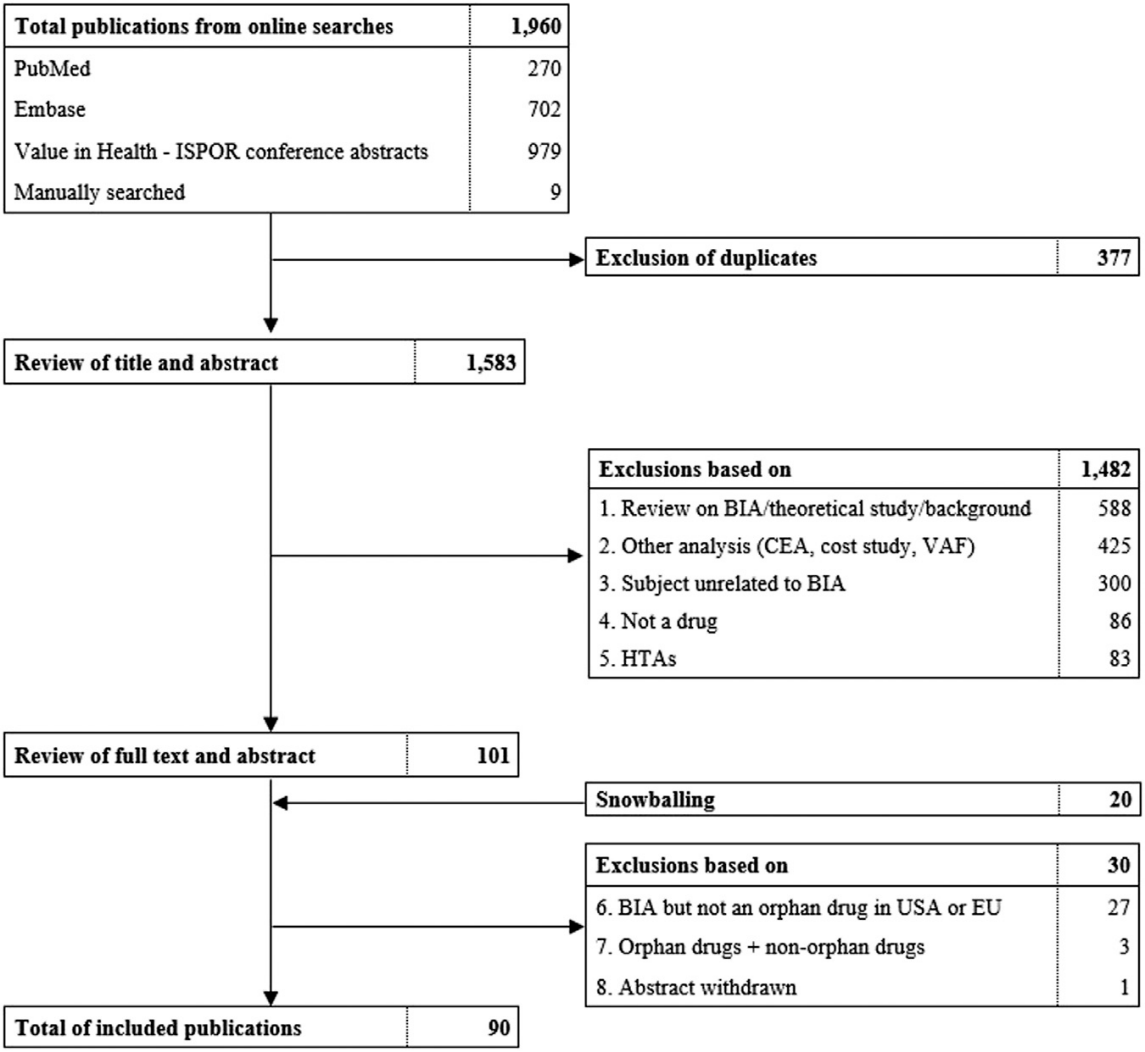
Flowchart of literature search and selection of publications. ISPOR, International Society for Pharmacoeconomics and Outcomes Research, BIA, budget impact analysis. CEA, Cost-effictiveness analysis. VAF, value assessment framework. HTA, health technology assessment.

This systematic review was conducted and reported in accordance with the Preferred Reporting Items for Systematic Reviews and Meta-Analyses (PRISMA) (Figure 1) (Liberati et al., 2009). A systematic search of peer-reviewed literature, ISPOR conference abstracts and gray literature was conducted in January 2020. PubMed and Embase were scanned for studies describing “budget impact analysis” and “orphan drugs”. A specific protocol and search strategy for every database was specified and is documented in Supplementary Material.

### Study Selection

Duplicate removal and filtering of studies was conducted in rayyan.qcri.org, a systematic review web tool. Two reviewers were involved in determining exclusion and inclusion criteria and final selection of studies. The first reviewer screened title and abstracts followed by a second round of examining abstracts and full texts. In case of uncertainties, the second reviewer decided on whether the study was included. This selection process was documented in a PRISMA flowchart (Figure 1).

### Eligibility Criteria

Studies analyzing the budget impact of one individual orphan drug or a combination of/multiple orphan drugs were considered. A study was included when the examined drug was labeled ‘orphan’ in the EU or the US for the indication described in the study. Language, country or year of publication were not considered exclusion criteria. Orphanet, European Medicines Agency (EMA) and U.S. Food and Drug Administration (FDA) official websites were used for verification of orphan drug status as of February 2020.

### Selection of Parameters and Data Retrieval

We adopted ISPOR good practice guidelines as quality standards (Mauskopf et al., 2007; Sullivan et al., 2014). Nine parameters were regarded as quality measures of the studies and consisted of; perspective, time horizon, intervention and comparator(s), target population, data sources, scope of costs, sensitivity analysis, discounting and validation.

Different publications concur that these parameters are essential for BIA (Trueman et al., 2001; Orlewska and Mierzejewski, 2004; Marshall et al., 2008; Orlewska and Gulacsi, 2009; Garattini and van de Vooren, 2011; Ferreira-Da-Silva et al., 2012; Sullivan et al., 2014; van de Vooren et al., 2014; Neyt et al., 2015; Faleiros et al., 2016; Mauskopf and Earnshaw, 2016, Mauskopf and Earnshaw, 2017; Silva et al., 2017; Ghabri et al., 2018; HIQA, 2018). Each parameter was subdivided into predefined categories (Table 1) that were attributed to a study if applicable. Data on categories for every study were summarized in a data extraction sheet in Excel (Supplementary Tables S2 and S3).

**TABLE 1:** Characteristics considered in methodological quality assessment of budget impact analyses.

Parameter	Perspective	Target population	Time horizon	Intervention	Comparator(s)	Scope of costs	Assumptions	Sensitivity analysis	Discounting	Validation	Data sources
Categories	Society,	Closed system (static),	Monthly,	Substitution,	No interventions,	Drug-only,	Population,	Deterministic univariate,	Yes,	Yes (+method),	Published literature,
	Third-party payer,	Open system (dynamic)	Quarterly,	Combination,	Current interventions,	Condition-related,	Intervention and/or comparator(s),	Deterministic multivariate,	No	No	Expert opinion,
	Hospital,		Annual +	Expansion	Off-label use of intervention	Other health care,	Costs,	Probabilistic			Market research,
	Patient,		Total years			Indirect	Treatment duration,				Historical data,
	Pharmacy,						Disease symptoms,				Data from other setting,
							Morbidity,				Registry,
							Mortality,				Claims database,
							Disease evolution,				Internal data from pharmaceutical company
							Other drug-related				

### Analysis

A descriptive analysis of all studies was carried out.

## Results

From the total of 1,960 publications initially identified, 90 were included in this systematic review (Figure 1; Supplementary Tables S2 and S3). Thirty-six (40%) studies originated from Europe, 27 (30%) from North-America, 12 (13%) from South-America and 15 (17%) from Asia. The budget impact of individual orphan drugs was analyzed in 69 studies, of which 12 (17%) were full text and 57 (83%) were conference abstracts. Our review covered a variety of orphan drugs targeting cancers (57%), blood disorders (13%), heart diseases (4%), endocrine diseases (7%), inflammation and immune disorders (3%), lung diseases (4%), genetic aberrations (6%), metabolic disorders (1%) and other diseases (4%). Of these drugs, 10% were designated for the treatment of ultra-rare diseases. Furthermore, BIAs of a combination of orphan drugs were identified in 21 studies, of which eight (38%) were available in full text and 13 (62%) as abstract.

### Individual Orphan Drugs

The characteristics of BIAs of individual orphan drugs are presented in Supplementary Table S2 and summarized in the following sections.

#### Perspective

One study considered a societal perspective and all other studies considered the budget holder perspective. With respect to the latter, one study calculated budget impact from the patient perspective, three studies considered the pharmacy perspective and the remaining studies considered the third-party payer perspective. Five studies considered two perspectives instead of one and five studies did not report the perspective of their analysis.

#### Target Population

In 17 (24.6%) studies, an open system with a dynamically changing population was considered. Thirty-seven (53.6%) studies utilized a closed system and described a static population. In one article (1.4%) both an open system and a closed system were described from different perspectives. Fourteen (20.3%) articles did not report on the dynamics of the eligible population.

#### Data Sources

All studies reported at least one or more data sources. Eligible patients and population size were based on; expert opinions (7.2%), published literature (26.1%), historical data (20.2%), registries (14.5%), market research (4.3%), internal data from pharmaceutical company (10.1%), claims database (15.9%) and data from other setting (11.6%).

Costs were retrieved from; expert opinions (11.6%), registries (30.4%), internal data from pharmaceutical company (14.5%), published literature (14.5%), data from other setting (23.2%) and claims databases (15.9%).

Intervention or comparator(s) (characteristics, treatment duration, resource use, market effects) were sourced from; expert opinions (18.8%), registries (7.2%), published literature (17.3%), internal data from pharmaceutical company (36.2%), data from other setting (10.1%), claims databases (2.9%) and market research (5.8%).

In one study (1.4%), discounting relied on data from another setting.

Three studies (4.3%) mentioned sources, without specification of the concerned inputs, such as; internal data from pharmaceutical company (4.3%), published literature (1.4%), claims database (1.4%), data from other setting (2.9%) and expert opinion (1.4%).

Four studies (5.8%) did not report on data sources.

#### Intervention and Comparator(s)

The majority of studies compared an orphan drug with one or more current interventions. In two studies, the orphan drug was compared to no intervention, as no current treatment was available. In five studies, a comparator was not specified or not reported. Introduction of the new intervention was mostly perceived as an additional alternative to current disease interventions in 44 studies, thus expanding the market. In 13 studies the new intervention was assumed to impact the market by substitution of the current treatments and in seven studies by combination with the current interventions. Some studies described the new intervention as influencing the market in multiple ways, on one hand by expansion and substitution, on the other hand by expansion and combination.

#### Scope of Costs

Drug-only costs were depicted in all studies. Of 47 studies that considered condition-related costs, three studies also addressed indirect costs of productivity loss.

#### Time Horizon

Apart from one study describing a 60-year horizon and seven references not reporting on a time horizon, all other references examined budget impact over a time horizon ranging from one to five years. In nine studies a one-year horizon was described whereas four references adopted a two-year time horizon. Twenty-eight references and two references respectively marked a three-year horizon and a four-year horizon. A five-year time horizon was considered in 20 references.

Furthermore, 48 studies reported their budget impact annually, seven studies reported monthly numbers and five references jointly reported monthly and annual calculations. In nine studies one net budget impact value over the total time horizon was mentioned.

#### Assumptions

Assumptions were made about the target population (size) in 56 studies, followed by assumptions about intervention and/or comparator(s) in 47 studies and costs in seven studies. The following assumptions were made: Treatment duration in six studies, morbidity in four studies, mortality in three studies and disease evolution in three studies. Discounting was assumed in one study and other drug-related assumptions in 24 studies. In ten studies budget impact assumptions were not reported.

#### Discounting

In 55 studies no information on discounting was found. ‘No discounting’ was explicitly mentioned in ten studies. In three studies, an annual discount rate was applied to the budget calculations. One study reported results with and without discounting.

#### Sensitivity Analysis

Deterministic univariate sensitivity analyses were performed in 14 studies. One study reported multivariate sensitivity analysis/scenario analysis while three other studies presented both univariate and multivariate sensitivity analyses. A probabilistic sensitivity analysis was carried out in one study. The use of a sensitivity analysis was mentioned in thirteen studies without further specification. Thirty-seven studies did not report any sensitivity analysis.

#### Validation

Four studies validated their results with either stakeholder opinions or comparison to a similar study. All remaining 65 studies did not corroborate their analysis.

### Combination of Orphan Drugs

The characteristics of BIAs for combination of orphan drugs are presented in Supplementary Table S3 and summarized in the following sections.

#### Perspective

In four studies budget impact was calculated from a hospital perspective. Pharmacy perspective was adopted alone in one study and in combination with hospital perspective in another study. In eight studies, third-party payer perspective was used and in another eight studies, the perspective was unspecified.

#### Target Population

This criterion was largely not applicable for combination of orphan drugs studies, apart from two studies describing a closed system population and one study an open system population.

#### Data Sources

Eligible patients and population size were retrieved from; registries (9.5%), published literature (19%), claims databases (33.3%), historical data (9.5%) and data from other setting (9.5%).

Costs were derived from published literature (38.1%), claims databases (52.4%), market research (9.5%), internal data from pharmaceutical company (19%), historical data (14.3%), registries (19%) and data from other setting (33.3%).

Intervention or comparator(s) (characteristics, treatment duration, resource use, market effects) were sourced from; registries (71.4%), claims databases (19.0%), published literature (19.0%), historical data (9.5%), internal data from pharmaceutical company (9.5%), market research (9.5%) and data from other setting (14.3%).

One study (4.8%) decided on discounting rate through published literature and one study (4.8%) did not report any data sources.

#### Scope of Costs

Drug-only costs were considered in all studies.

#### Time Horizon

In five studies, a one-year horizon was considered for budget impact analysis. One study used a two-year horizon, four studies used a five-year horizon and two studies used a six-year horizon. In one study a seven-year horizon was considered.

One other study considered a nine-year horizon, two studies a ten-year horizon and two other studies a 12-year horizon consisting of a six-year retrospective and a four-year prospective analysis. One study calculated their budget impact based on a 17-year time horizon, one on a 20-year horizon consisting of both ten-year retrospective and prospective analyses, and one on a 21-year horizon consisting of a 13-year retrospective and eight-year prospective analysis.

Annual calculations were reported consistently except for one study reporting monthly values, another one reporting one net value and one study reporting annual and monthly values.

#### Assumptions

One study made assumptions about costs and three studies about the target population. Ten studies made assumptions about the interventions (market effects) and one study about treatment duration. Eleven studies made other drug-related assumptions whereas nine studies did not mention any assumptions.

#### Discounting

Majority of studies did not report on discounting except for three that explicitly mentioned ‘no discounting’ and one applying a 3.5% discount rate on their drug costs.

#### Sensitivity Analysis

A total of six studies reported on sensitivity analyses; three studies conducted deterministic univariate sensitivity analysis, two studies accounted for structural uncertainty through deterministic multivariate sensitivity analyses and one study combined univariate and multivariate sensitivity analyses.

#### Validation

Five studies validated their data inputs through comparison with reliable data sources. Nine studies compared their budget impact outcomes with a similar study in the same or different region; of these nine studies, two also performed a statistical validation. Seven studies did not report on validation of their analysis.

## Discussion

In this review, the methodological quality of BIAs for orphan drugs was systematically assessed based on expert-composed ISPOR good practice guidelines. Majority of our studies covered orphan drugs targeting cancer or blood disorders and primarily originated from Europe and North-America. By mapping out the geographic distribution of BIAs, our review suggests that BIAs are more frequently performed in high-income countries and the Anglosphere than in low- and middle-income countries. We determined that as per ISPOR recommendations, most studies take on a third-party payer perspective, time-horizons are mainly set to one-to-five years while budget impacts are reported periodically, discounting is scarcely applied and overall, data is collected from experts, literature, historical or epidemiological data, national registries, claims databases, market research or pharmaceutical companies. Contrary to ISPOR recommendations, numerous studies do not appropriately account for a dynamically changing population, although a static population may be considered when the population is very defined, scope of costs is often limited to drug-only costs, and analyses are rarely validated. Furthermore, all studies require several assumptions, however these assumptions were seldomly justified nor sufficiently tested by sensitivity analyses. This perceived shortage of sensitivity analyses, may be subject to the numerous BIAs depicted in forms of abstracts which merely capture summarized analyses. Compared to full text BIAs, abstracts described fewer sensitivity analyses, however, in both text types these analyses were often limited when included. Additionally, high-income countries generally performed more and elaborated sensitivity analyses than low- and middle-income countries. Our review shows that most assumptions are made of population size and intervention/comparator(s) market uptake. Due to lack of readily available data and sources specifically on orphan drugs, resorting to assumptions is disadvantageous but inevitable. A sensitivity analysis is essential to investigate the influence of assumptions on structural aspects or variable inputs of the BIA. Moreover, sensitivity analyses allow a more comprehensive prediction of budget impact which should be a staple for orphan drugs considering their data scarcity. This review corroborates precedent findings (Mauskopf et al., 2005; Orlewska and Gulacsi, 2009; van de Vooren et al., 2014; Faleiros et al., 2016) on substandard quality and heterogeneity of BIAs. Multiple factors might be at the cause of the low quality of existing BIAs. One factor is that BIA remains a fairly new method. Not until the nineties did jurisdictions begin requesting BIAs as part of reimbursements and the first methodological guidelines were written only at the end of that decade (Mauskopf, 1998; Sullivan et al., 2014). Since then, the concept of a BIA has evolved with expanded, detailed and updated recommendations were published lastly by the health expert community of ISPOR in 2014 (Sullivan et al., 2014).

Nonetheless, our review assessed only ten and five studies of individual and a combination of orphan drugs, respectively, that were released prior to these ISPOR guidelines (Supplementary Tables S2 and S3).

This implies that, even with updated ISPOR guidelines available, majority of studies still fail to meet methodological quality standards. Another contributor to improper quality might be that, in the past, there have been counterarguments on the usefulness of BIAs due to the close proximity of the technique to CEA (Niezen et al., 2009). However, BIA allows to gage how the total budget in a certain health demographic changes after the introduction of an intervention making this a powerful tool to predict affordability (Garattini and van de Vooren, 2011; Sullivan et al., 2014; Mauskopf and Earnshaw, 2017; Pearson, 2018). Hence, BIAs have become, commensurate with economic evaluations, established as a prerequisite for reimbursement applications in many jurisdictions (Pearson, 2018). As orphan drugs are taking up an emergent share in pharmaceutical expenditure but health care resources remain scarce, correctly predicting budgetary impact is fundamental (Mestre-Ferrandiz et al., 2019). This could facilitate and sustain market access of orphan drugs, which may otherwise be neglected, in a certain health setting.

### Recommendations for Future Budget Impact Analyses for Orphan Drugs

Future analyses should be rigorously assessed on adherence to guidelines of methodology and reporting of a budget impact (i.e. national guidelines or ISPOR good practice guidelines) before it is handed to the appropriate budget holder and/or published. We offer a concise template (Table 1) that is required for a comprehensive and correct budget impact analysis and to which budget holders can refer to when reviewing a reimbursement application. All relevant perspectives, being one or more budget holders, should be adopted in the analysis. A dynamic target population is one that accounts for influx and efflux of patients throughout the selected time frame. Costs should not be restricted to drug-costs but also consider condition-related costs such as administration and adverse event costs. To determine the influence of uncertainties on budget impact, assumptions should consistently be accompanied by exhaustive sensitivity analyses. Validation after conducting a budget analysis should be urged and could be done by consulting budget holders and corroborating model parameters. Recently, a study by (Geenen et al., 2020a) proposed a novel method of BIA that could be useful for validation. Additionally, all inputs and formulas should be validated by a second budget impact expert. Comparing the outcomes from earlier analyses with the current budget impact of orphan drugs could deliver clarity on future design or adjustments to budget impact analyses. Furthermore, methodological choices in a BIA should continuously be motivated. Finally, an active effort toward facilitating BIAs should be made by systematically collecting and transparently publishing the required data on orphan drugs.

### Strengths and Limitations

This review is the first to methodologically assess the quality of BIAs specifically for orphan drugs. The primary limitation of the current literature on BIAs of orphan drugs is that the majority of studies are reported in the form of conference abstracts, which provide limited information about the objectives, design and results of the study. However, as the domain is still evolving, adding abstracts to this review allowed us to analyze the quality of most recent data on BIA for orphan drugs. This review thus includes 83% and 62% abstract-only BIAs for individual and a combination of orphan drugs, respectively. It should also be noted that BIAs directly submitted to reimbursement agencies were not studied.

## Conclusion

With the rise of orphan drug expenditure, budget impact analysis forms an important tool to assess affordability of adopting a new orphan drug in a certain health setting. This research finds that budget impact analyses on orphan drugs are of poor-quality and have low methodological adherence to standardized good practice guidelines. Continued improvement of the validity of these analyses should be prioritized. Future studies should be directed toward; multiple perspectives, an open population, costs beyond drug-only costs, exhaustive sensitivity analyses and validation of their budget impact framework.

## Data Availability

The original contributions presented in the study are included in the article/Supplementary Material, further inquiries can be directed to the corresponding author.
