# Supplementary material for: Methodological Quality Assessment of Budget Impact Analyses for Orphan Drugs: A Systematic Review
Source: Front Pharmacol. 2021 Apr 21;12:630949. doi: 10.3389/fphar.2021.630949 (PMC8098807; doi:10.3389/fphar.2021.630949)
Supplement: Supplementary file 1 [file table1.docx]

**Supplementary Table 1.** Characteristics of budget impact analyses for individual orphan drugs*

| Parameter | Intervention | Data sources | Perspective | Target population | Time horizon | Comparator(s) | Scope of costs | Results of budget impact analysis | Sensitivity analysis | Validation | Comments and limitations |
| --- | --- | --- | --- | --- | --- | --- | --- | --- | --- | --- | --- |
| *(Al Jedai et al., 2019),*  *Saudi-Arabia* | emicizumab  –  expansion | expert opinion | third-party payer | closed system (static) | not reported – 5 years | current intervention | drug-only, condition-related, indirect | scenario 1 without emicizumab:   - total 5-year costs: SAR 186.0 million   scenario 2 with emicizumab:   - total 5-year savings (%) in total and average patient costs: 21.5% - total 5-year costs: SAR 146.3 million   lower: bleeding rates, administration costs, travel costs, hospitalizations and surgeries  drug acquisition costs contribute over 75% of the total costs | base-case scenario: 100% of patients switch to emicizumab   - sensitivity analysis varied inputs with 20% | not reported | not reported: calculations and results sensitivity analysis, calculations for total costs and budget savings, limitations  limited sources consulted |
| *(Alexandre et al., 2017),*  *Brazil* | crizotinib  –  expansion | published literature, registry | third-party payer | closed system (static) | annual –  3 years | current interventions | drug-only | - costs of treatments with crizotinib (year 1 - year 2 - year 3): BRL34.722.255 - BRL37.847.258 - BRL41.253.511 - costs of treatments without crizotinib (year 1 -year 2 - year 3): BRL1,736,136 - BRL1,892,388 - BRL2,062,703   incremental budget impact over 3 years: BRL5,691,227 (5% of lung cancer patients) or BRL0.07 PMPM | not reported | not reported | simplistic  limitations not reported |
| *(Alva et al., 2015),*  *Mexico* | carfilzomib  –    expansion | published literature, historical data, expert opinion | third-party payer | not reported | monthly –  5 years | current intervention | drug-only, condition-related | average budget increase with 5% uptake every year: 0.0033% | not reported | not reported | simplistic  limitations not reported |
| *(Alva et al., 2018),*  *Mexico* | blinatumomab  –  substitution | published literature,  registry | third-party payer | closed system (static) | annual –  5 years | current interventions | drug-only | average variation per year:   - decrease, -0.001% (group with less than 1-year olds) - 0.01% (high risk group) - 0.014% (very-high risk group) - decrease, -0.029% (standard risk group) | sensitivity analysis suggests robust results  with variations of:  0.002% (group with less than 1-year olds)  0.04% (high risk group)  0.047% (very-high risk group)  - 0.003% (standard risk group) | not reported | budget impact reported as percentages and no detail on the actual amount in Mexican pesos, name of comparators not mentioned, no detail on sensitivity analysis, limitations not explicitly mentioned, no data on number of patients |
| *(Appukkuttan et al., 2019),*  *USA* | copanlisib  –  expansion | market research,  expert opinion, published literature, internal data from pharmaceutical company, data from other setting, historical data | third-party payer | closed system (static) | monthly –  1 years | current interventions,  off-label use of intervention | drug-only, condition-related, population, intervention, costs | without copanlisib   - plan total cost: $1,612,062 - total budget impact ($PMPM): $0.11   with copanlisib   - plan total cost:   $1,369,421: copanlisib $92,043  (increase market share off-label use drugs OB and LR with resp., 9.0% to 13.1% and 0.3% to 12.0%)   - cost increase:   $238,536 (drug acquisition)  $3,565 (drug administration and prophylaxis)  $539 (monitoring cost)   - total budget impact increase:   $242,641 (21.8% from copanlisib, 12.9% from OB and 65.3% from LR)   - total budget impact ($PMPM):   $0.13 (increase of $0.02) | most sensitive to number of eligible patients and drug costs  total budget impact ranged from $181,980 - $303,301 or PMPM: $0.015 - $0.025: | not reported | limitations: no other perspectives such as Medicare or societal perspective, assumptions, not accounted for cost differences, not necessarily generalizable to real-world practices  inflated estimates of off-label use  longer time horizon needed for changes in budget impact over time |

| Parameter | Intervention | Data sources | Perspective | Target population | Time horizon | Comparator(s) | Scope of costs | Results of budget impact analysis | Sensitivity analysis | Validation | Comments and limitations |
| --- | --- | --- | --- | --- | --- | --- | --- | --- | --- | --- | --- |
| *(Avxentyev et al., 2018),*  *Russia* | nilotinib  –  expansion | internal data from pharmaceutical company | third-party payer | closed system (static) | monthly –  4 years | current interventions | drug-only, condition-related | - cumulative 4-year budget spending:   US$ 12.4 million less compared to dasatinib   - monthly medication costs:   US$2,626 (= $54/2.0% less than dasatinib and US$1,011/27.8% less than bosutinib)   - total 4-year medical costs:   US$70,336 (= 13.9% less than dasatinib and 37.3% less than bosutinib) | not reported | not reported | calculations not specifically with and without nilotinib, no limitations, no sensitivity analysis, limited information available  sources on costs not reported |
| *(Barbieri et al., 2014),*  *Italy* | ambrisentan  –  substitution | expert opinion, internal data from pharmaceutical company | third-party payer | open system (dynamic) | not reported –  3 years | current intervention | drug-only, condition-related | ambrisentan:   - total 3-year cost: € 86,482,146 - 3-year cost savings: € 1.1 million   bosentan:  total 3-year cost: € 87,594,291 | sensitivity analysis corroborated base case findings | yes (expert opinion) | simplistic  not reported: limitations, sources on costs and population (size) |
| *(Bergamelli Ramos et al., 2019),*  *Brazil* | regorafenib  –  expansion | registry,  claims database, published literature, internal data from pharmaceutical company,  data from other setting | third-party payer | open system (dynamic) | annual –  5 years | no intervention | drug-only, condition-related | scenario 1: only best supportive care    scenario 2: market-share from 70% to 100%   - first-year costs: BRL 5 million - overall 5-year costs: BRL 41 million | most sensitive parameters: proportion of patients treated with sorafenib and patients eligible for 2nd line treatment | not reported | simplistic  limitations not reported |
| *(Bhardwaj et al., 2008),*  *Canada* | sorafenib  –  expansion | claims database, historical data | third-party payer | not reported | annual –  3 years | not reported | drug-only | financial impact in CAD$  (2008 - 2009 – 2010):    $3.7M - $7.1M - $9.7M | not reported | not reported | simplistic  limitations not reported |
| *(Bharmal et al., 2019),*  *USA* | avelumab  –  expansion | published literature, internal data from pharmaceutical company, registry,  data from other setting, historical data | third-party payer | closed system (static) | annual –  3 years | current interventions | drug-only, condition-related | without avelumab   - total 3-year cost:   $11,710,115 or $0.0109 PMPM  with avelumab   - total 3-year cost:   $9,066,942 or $0.0084 PMPM   - cumulative budget impact or total savings:   $2,643,173 or $0.0025 PMPM or  22.6% change in budget | most sensitive parameters are treatment duration for 1L pembrolizumab and nivolumab:   - +300% ‣ total budget impact is -$7,961,329 or -$0.0074 PMPM - -75% ‣ total budget impact is $2,146,493 or $0.0020 PMPM | not reported | treatment duration assumed to be the same for both immuno-oncology agents, median value of progression free survival is used as a proxy for treatment duration –  robustness of assumptions is limited by the availability of data –  updated list of comparators and additional country-specific analysis should be carried out –  assumed that introduction of avelumab only alters market shares of immuno-oncology agents and not of chemotherapies –  patient-to-patient variance was not incorporated in the model –  only one therapy per patient –  large driver of cost difference due to gap between median progression free survival of avelumab and pembrolizumab trial data –  increased survival of patients should result in increased costs |

| Parameter | Intervention | Data sources | Perspective | Target population | Time horizon | Comparator(s) | Scope of costs | Results of budget impact analysis | Sensitivity analysis | Validation | Comments and limitations |
| --- | --- | --- | --- | --- | --- | --- | --- | --- | --- | --- | --- |
| *(Brosa et al., 2011), Spain* | somatuline autogel  –  expansion, substitution | registry,  claims database | not reported | closed system (static) | annual –  not reported | current intervention | drug-only, condition-related | somatuline autogel   - overall treatment cost per patient/per year: € 11,857 - mean cost savings (30% of patients benefit of extended dose interval):   € 2,019   - reduction of treatment cost (if patient treated increases from 50% to 70%, respectively):   € 0.3 million to € 1.2 million  sandostatin LAR   - overall treatment cost per patient/per year: € 12,165   sandostatin LAR has higher acquisition and administrative costs than somatuline autogel | multivariate/scenario:  different scenarios based on the proportion of patients using different dose ranges | not reported | simplistic  no sensitivity analysis and limitations not reported |
| *(Brosa et al., 2015), Spain* | mifamurtide  –  combination | registry, claims database, internal data from pharmaceutical company, data from other setting | third-party payer | open system (dynamic) | annual –  60 years | current interventions | drug-only costs | - cost per treatment:   €101,388 - €124,785   - yearly cost (10% - 100% of potential patients): €671,000 - €6.7 million - yearly cost considering the number of vials: €0.5 - €5 million - pharmaceutical expenditure:   €2.5 million - €3.4 million  Mifamurtide use in half of potential patients  ‣ 1.3% of pharmaceutical spending on orphan drugs and 0.03% of total pharmaceutical expenditure | model results mostly influenced by time horizon and discount rate | not reported | open system but only considered influx of patients and not efflux of patients  annual discount rate of 3% was applied  no limitations reported, only maximum of yearly costs reported and the cumulation of total costs over 60 years |
| *(Carlton et al., 2018),*  *USA* | deflazacort  –  expansion | published literature, registry, internal data from pharmaceutical company | two perspectives: third-party payer | closed system (static) | annual –  3 years | no intervention | drug-only, condition-related | Year 1 - Year 2 - Year 3   - pharmacy cost (PMPM): $0.008 - $0.012 - $0.0116 - budget impact savings: $19,419/$0.002PMPM -$29,001/$0.002PMPM - $38,584/$0.003PMPM - budget impact savings with Medicaid rebate: $43,518/$0.004PMPM - $64,990/$0.005PMPM - $86,464/$0.007PMPM   3-year cumulative total   - budget impact savings: $87,004/$0.002PMPM - budget impact savings with Medicaid rebate: $194,972/$0.005PMPM   total annual cost per patient with deflazacort was  ≈ $12,000 less than patients who are not treated (commercial population) | not reported | not reported | no active comparator  time horizon does not capture long-term benefits  published source information may not be accurate  indirect costs such as caregiver burden or family loss are not included |
| *(Cristino et al., 2015), Belgium* | denosumab  –  expansion | published literature,  historical data, internal data from pharmaceutical company | third-party payer | not reported | annual –  3 years | current intervention | drug-only | denosumab   - total drug expenditure: € 550,940 - total drug savings: € 409,372 - 3-year budget impact on the overall health care budget: € 141,372   74% of denosumab expenditure offset by its clinical benefit | not reported | not reported | simplistic  full clinical benefit of denosumab not considered |

| Parameter | Intervention | Data sources | Perspective | Target population | Time horizon | Comparator(s) | Scope of costs | Results of budget impact analysis | Sensitivity analysis | Validation | Comments and limitations |
| --- | --- | --- | --- | --- | --- | --- | --- | --- | --- | --- | --- |
| *(Demir et al., 2018), Turkey* | nintedanib  –  expansion | data from other setting, claims database, registry,  historical data | third-party payer | closed system (static) | annual –  3 years | current intervention | drug-only | - savings (in Turkish lira) on IPF drug expenditure and pirfenidone   (year 1 - year 2 - year 3):  5,441,698L - 8,528,165L - 9,053,578L   - Overall 3-year savings (in Turkish lira) on IPF drug expenditure and pirfenidone: 23,023,441L   nintedanib market shares from overall IPF market (year 1 - year 2 - year 3):  33.7% - 43.0% - 43.0% | not reported | not reported | budget impact calculations with and without nintedanib not clearly stated  limited information  not reported: sensitivity analysis, limitations, specific population size number |
| *(Derkach et al., 2016),*  *Russia* | obinutuzumab and ibrutinib  –  expansion | registry, expert opinion, internal data from pharmaceutical company | third-party payer | open system (dynamic) | annual –  3 years | current interventions | drug-only | scenario 1 (obinutuzumab and ibrutinib):  year 1 - year 2 - year 3:  € 22.200 - € 40.400 - € 66.900  scenario 2 (obinutuzumab) compared to scenario 3 (ibrutinib):  year 2: obinutuzumab is € 6.600 (12.4%)  year 3: € 33.100 (41.5%) lower than ibrutinib | not reported | not reported | simplistic  limitations not reported  no specifics on data sources |
| *(Djambazov et al., 2018),*  *Bulgaria* | inotuzumab ozogamicin  –  expansion | historical data,  registry | third-party payer | not reported | annual –  5 years | not reported | drug-only, condition-related | scenario 1:   - budget savings (year 1 – year 5):   4,917 BGN - 14,750 BGN  cumulative 5-year budget savings:  49,168 BGN | not reported | not reported | simplistic  limitations not reported |
| *(Fan et al., 2018),*  *USA* | nintedanib  –  substitution | published literature, claims database, internal data from pharmaceutical company, data from another setting | third-party payer | closed system (static) | annual –  2 years | current intervention | drug-only, condition-related | Year 1:  50% nintedanib and 50% pirfenidone   - total cost savings: $3,439 - impact on cost per member per month: $0.00 - impact on cost per treated patient per month: decrease -$4.81   Year 2:  55% nintedanib and 45% pirfenidone   - total cost savings: $16,907 - impact on cost per member per month:   decrease -$0.001  impact on cost per treated patient per month: decrease -$20.32 | variation of +/- 0.005 suggests results are robust | not reported | simplistic, very concise  sensitivity analysis not corroborated with hard numbers limitations not reported  data sources not specified per parameter |
| *(Fust et al., 2017),*  *Sweden* | telotristat ethyl  –  combination | published literature,  claims database,  internal data from pharmaceutical company | not reported | open system (dynamic) | annual –  5 years | current intervention | drug-only | telotristat ethyl + standard of care   - net 5-year budget impact:   € 11,898 or less than € 4.88 per patient per month in each year of the analysis | not reported | not reported | simplistic  no sensitivity analysis and limitations not reported |
| *(Germanyuk et al., 2017), Ukraine* | somatropin  –  not reported | registry, claims database,  expert opinion | third-party payer | closed system (static) | annual –  not reported | current interventions | drug-only | - cost percentage of population salary: 44.1% - 370.6% - yearly budget impact of somatropin analogues: 20965289.75 - 176002076.6 UAH - yearly budget saving according to cheapest analogue:   155036786.85 UAH  somatropin therapy is economically inaccessible for Ukrainian population | not reported | not reported | very simplistic  not transparent about the calculations of budget impact per somatostatin analogue  comparison between analogues not effectively done  limitations not reported |

| Parameter | Intervention | Data sources | Perspective | Target population | Time horizon | Comparator(s) | Scope of costs | Results of budget impact analysis | Sensitivity analysis | Validation | Comments and limitations |
| --- | --- | --- | --- | --- | --- | --- | --- | --- | --- | --- | --- |
| *(Hollmann et al., 2018a),*  *England* | tisagenlecleucel  –  expansion | published literature,  market research,  registry,  internal data from pharmaceutical company | third-party payer | closed system (static) | annual –  3 years | current interventions | drug-only, condition-related | scenario 1: without tisagenlecleucel  scenario 2: with tisagenlecleucel   - year 1 - year 2 - year 3:   £ 570,210 - £ 11,495,429 –  £ 14,484,241  cumulative 3-year budget impact:  £ 26,549,880 | not reported | yes (United Kingdom clinicians) | simplistic  limitations not reported |
| *(Hollmann et al., 2018b),*  *England* | tisagenlecleucel  –  expansion | published literature,  data from other setting,  internal data from pharmaceutical company,  registry | third-party payer | open system (dynamic) | annual –  3 years | current interventions | drug-only, condition-related | with tisagenlecleucel   - budget impact (year 1 - year 2 - year 3):   £221,456 - £4,464,553 - £5,625,337   - cumulative 3-year budget impact: £10,311,346   tisagenlecleucel in England is less than £6 million annually and well below the £20 million NHS budget impact threshold | not reported | not reported | no sensitivity analysis, did not consider how many patients would stop treatment because of for example, side effects  no limitations reported  calculations of budget impact not explicitly stated  limited information available |
| *(Jain et al., 2019),*  *USA* | lanadelumab  –  expansion | historical data,  internal data from pharmaceutical company | third-party payer | open system (dynamic) | annual –  3 years | current intervention | drug-only, condition-related | scenario 1: with lanadelumab  scenario 2: without lanadelumab  Total annual savings   - in 2018: 15% of HAEI/II patients on lanadelumab –   $289,878 or $17,019 per patient per year or  $0.024 PMPM   - in 2020: 40% of HAEI/II patients on lanadelumab –   $914,779 or $52,175 per patient per year or $0.076 PMPM | +/- 10 variation of base value for each parameter results in PMPM savings of $0.020 to $0.029 for year 2018 and  $0.067 to $0.089 for year 2020 | not reported | simplistic  assumptions about prophylaxis uptake  no savings for year 2019 were reported |
| *(Kawalec et al., 2010),*  *Poland* | ambrisentan  –  expansion | registry, claims database,  internal data from pharmaceutical company | third-party payer | not reported | annual –  5 years | current interventions | drug-only | scenario 1: bosentan, iloprost, treprostinil or silendenafil  scenario 2: added ambrisentan   - year 1: 178,782 PLN (64,719 PLN - 342,497 PLN) - year 2: 385,054 PLN (132,673 PLN - 769,277 PLN) - year 3: 614,006 PLN (203,224 PLN - 1,266,372 PLN) - year 4: 646,137 PLN (206,854 PLN - 1,366,336 PLN) - year 5: 674,573 PLN (210,053 PLN - 1,455,310 PLN) | not reported | not reported | simplistic  limitations not reported |
| *(Knoth et al., 2018),*  *USA* | lenvatinib  –  substitution | internal data from pharmaceutical company | third-party payer | closed system (static) | monthly –  1 year | current interventions | drug-only, condition-related | baseline year chemo without lenvatinib:   - total annual uHCC treatment costs: $4.04 million - Cost savings: $0.01PMPM   comparator year chemo with lenvatinib:  total annual uHCC treatment costs: $3.92 million | most sensitive to changes in acquisition costs of lenvatinib and sorafenib and cost of best supportive care | not reported | simplistic,  short time horizon  static population  limitations not reported |

| Parameter | Intervention | Data sources | Perspective | Target population | Time horizon | Comparator(s) | Scope of costs | Results of budget impact analysis | Sensitivity analysis | Validation | Comments and limitations |
| --- | --- | --- | --- | --- | --- | --- | --- | --- | --- | --- | --- |
| *(Kohli et al., 2017),*  *USA* | telotristat ethyl + ocreotide LAR  –  combination | published literature,  claims database,  internal data from pharmaceutical company | third-party payer | closed system (static) | annual –  3 years | current intervention | drug-only | net formulary budget impact  (year 1 – year 2 – year 3): $1,474,000/$0.01PMPM - $1,837,000/$0.02PMPM - $2,312,000/$0.02PMPM | most sensitive parameters are estimation of average ocreotide LAR and proportion of CS patients progressing to uncontrolled carcinoid syndrome diarrhoea | not reported | simplistic  limitations not reported |
| *(Kolbin et al., 2018),*  *Russia* | obinutuzumab  –  expansion, combination | not reported | third-party payer | closed system (static) | not reported –  3 years | current interventions | drug-only | - total resource-saving with obinutuzumab within 3 years: 635 547 EUR - obinutuzumab usage up to 10%, 20% and 30%,   costs decrease by 2.7%, 2%, and 2.3%  ‣ introduction of obinutuzumab+bendamustine is economically feasible in the Russian Federation healthcare system | not reported | not reported | very little information,  very concise analysis  budget impact numbers not clearly stated with and without obinutuzumab per year or per month,  no limitations reported,  insufficiently transparent about data sources |
| *(Kulikov et al., 2015),*  *Russia* | dasatinib  –  expansion, substitution | registry | third-party payer | not reported | annual –  1 years | current interventions | drug-only, condition-related | scenario 1: 100% patients high-dose imatinib  scenario 2: % patients from high-dose imatinib to dasatinib:   - budget savings: 463 milion rubles or $7.7 milion or 10% of actual CML budget in Russia - annual treatment cost: 1,720,111 rubles or $28,365   ‣ 100% of imatinib-resistant patients can be provided with dasatinib as a second-line treatment without increase of the total national CML budget | not reported | not reported | simplistic  no sensitivity analysis and limitations not reported |
| *(Lee et al., 2018),*  *Finland* | pasireotide LAR  –  expansion | published literature, expert opinion | societal perspective | open system (dynamic) | annual –  5 years | not reported | drug-only, condition-related, indirect | year 1 (2018)   - incremental budget impact: €45,247 or   2% of total expenditure  year 5 (2022)   - incremental budget impact:   € 231,318 or 9% of total expenditure  ‣ higher treatment costs offset by lower indirect and adverse event costs | sensitivity analysis suggests that budget impact is robust | not reported | open system but only considered influx of new patients, not efflux of patients that do not meet criteria anymore  limited information reported  only first year and fifth year budget impact reported and nothing about years two, three and four |
| *(Lin et al., 2019),*  *USA* | patisiran  –  expansion | not reported | two perspectives: third-party payer | closed system (static) | annual –  3 years | current interventions | drug-only, condition-related | scenario 1: reimbursement of inotersen  scenario 2: reimbursement of patisiran and inotersen  commercial health plan perspective   - 3-year incremental budget impact:   $0.008 PMPM   - total incremental costs: $284,449   Medicare Advantage perspective   - 3-year incremental budget impact: $0.002 PMPM | not reported | not reported | very concise, limited information  eligible population in the hypothetical plan not mentioned  no sensitivity analysis  insufficient transparency about the assumptions |

| Parameter | Intervention | Data sources | Perspective | Target population | Time horizon | Comparator(s) | Scope of costs | Results of budget impact analysis | Sensitivity analysis | Validation | Comments and limitations |
| --- | --- | --- | --- | --- | --- | --- | --- | --- | --- | --- | --- |
| *(Lorenzoni et al., 2018),*  *Italy* | efmorococtog alfa (rFVIIIFc)  –  expansion | published literature,  data from other setting, expert opinion, registry | third-party payer | open system  (dynamic) | annual –  3 years | current interventions | drug-only costs | current environment with conventional FVIII   - total 3-year cost: €555,277,691     new environment with rFVIIIFc   - total 3-year cost: €541,897,466 - total 3-year savings: €13,380,255   or 2.4% | Univariate results are robust but most sensitive to dosage of conventional products:  -/+30% -> €20million extra costs/€30 million savings  and rFVIIIFc: -/+30% -> €30 million savings/€20 million extra costs  and product costs:lower price conventional products -> savings decreased to €1million  Scenario:  1) expanded population -> savings €18,197,391or 2.4%  2) switch to prophylaxis treatment: savings €2,654,595 or 0.4% | not reported | Lack of sufficient data on treatment adherence, parameters were extrapolated from other studies |
| *(Masoura et al., 2013),*  *Greece* | deferasirox  –  expansion | internal data from pharmaceutical company | third-party payer | closed system (static) | not reported –  5 years | current interventions | drug-only, condition-related | without deferasirox   - acquisition costs: €3,545,406 - administration costs: €11,889,133   with deferasirox   - acquisition cost: €5,738,323 (+62%) - administration costs: €4,993,474 (-58%)   total 5-year expenditure savings:  €4,702,742 or 30% | Type of sensitivity analysis not specified, no results reported | not reported | avoidance of administration costs for infused treatments could offset higher acquisition cost for deferasirox |
| *(McMullen et al., 2017),*  *USA* | efmorococtog alfa -recombinant human Factor VIII Fc fusion protein (rFVIIIFc)  –  expansion | registry, internal data from pharmaceutical company,  market research,  published literature,  registry | third-party payer | open system (dynamic) | annual –  2 years | current interventions | drug-only | year 1 - year 2   - impact on budget: US$122,107 – US$112,126 - impact on budget (%): 1.4 - 1.3 - cost per patient: US$6,188 – US$5,682 - cost per bleed avoided: US$1,974 – US$1,808 - cost per member: US$0.12 – US$0.11   total (across 2 years)   - budget increase: US$ 243,233 - budget increase: 1.4% - cost per patient: US$11,869 - cost per bleed avoided: US$1,891 - cost per member: US$0.23   with rFVIIIFc   - episodic budget: US$1,793,080 - prophylaxis budget: US$6,975,144 - total private payer budget: US$8,768,224   without rFVIIIFc   - episodic budget: US$2,044,868 - prophylaxis budget: US$6,611,229 - total private payer budget: US$8,656,098 | scenarios:  scenario 1: episodic to episodic with and without rFVIIIFc  scenario 2: episodic to prophylaxis with and without rFVIIIFc  scenario 3: prophylaxis to prophylaxis with and without rFVIIIFc  ‣ most sensitive to switch from episodic to prophylaxis: 100% switch then budget impact +37.7% or 0% switch then budget impact  -1.6% | no | limitations: switching patterns based on market research data, non-reflective of real-world switching behaviour |
| *(Mucha et al., 2015),*  *Poland* | dasatinib  –  expansion | historical data | third-party payer | closed system (static) | annual –  2 years | current intervention | drug-only, condition-related | scenario 1: with dasatinib   - increase in expenses   (year 1 – year 2):  €127,279 - €189,224  scenario 2: without dasatinib | not reported | not reported | additional costs excluded, simplistic, no sensitivity analysis,  not reported: limitations, data sources on costs and other inputs |

| Parameter | Intervention | Data sources | Perspective | Target population | Time horizon | Comparator(s) | Scope of costs | Results of budget impact analysis | Sensitivity analysis | Validation | Comments and limitations |
| --- | --- | --- | --- | --- | --- | --- | --- | --- | --- | --- | --- |
| *(Nalysnyk et al., 2018),*  *USA* | eliglustat  –  substitution | internal data from pharmaceutical company,  claims database, registry | third-party payer | closed system (static) | annual –  3 years | current interventions | drug-only, condition-related | current market:   - annual total cost: $11,210,981 - total savings:   $1,526,710 or 13.6% or  0.025 $PMPM   - 3-year total cost: $33,632,943 - 3-year total savings: $4,580,130 or 13.6%   new market   - annual total cost: $9,684,271 - 3-year total cost: $29,052,813 | structural uncertainty:  scenario 1: maturing shift to eliglustat  50% → -15.4% or  -0.029 $PMPM  100% → -22.9% or  -0.043 $PMPM  scenario 2: site of care management  10% → -8.9% or -0.014 $PMPM  scenario 3: conservative mark-up  20% all sites →  -10.7% or -0.018 $PMPM  ‣ parameter uncertainty: increase in average weight and dose →  -$7,092,599 or -17.7%  adherence →  -$5,002,699 or -13.7% | no | limitations: generalizability is limited, assumed payers responsible for all costs, no co-payment and coinsurance considered, no analysis of safety/efficacy/outcomes, not accounted for poor metabolizers |
| *(Naranjo et al., 2017),*  *Mexico* | blinatumomab  –  expansion | published literature | third-party payer | not reported | not reported –  5 years | current interventions | drug-only, condition-related | blinatumomab   - total 5-year increment from $3.5 billion healthcare budget: 0.14981% or an average cost of $5,204,192 - cost per response/effectively treated patient: $179,129 - cost of resource use: $130,764   FLAG-IDA   - cost per response/effectively treated patient: $262,455   cost of resource use: $83,985 | not reported | not reported | simplistic  no sensitivity analysis and limitations not reported |
| *(Paladini et al., 2012),*  *Brazil* | azacytidine  –  expansion | published literature, data from other setting | third-party payer | closed system (static) | not reported –  3 years | current intervention | drug-only, condition-related | - total 3-year budget savings:   BR$85,000 or US$45,000 compared to decitabine  50% treatment uptake → budget impact:  BR$45 million or US$25 million | results and type of sensitivity not reported | not reported | no specifics on data sources or sensitivity analysis  only one cumulative net budget impact reported not yearly/monthly results  number of patients not reported, no details on calculations of budget impact, very limited information  limitations not reported |
| *(Paiva and Asano, 2016),*  *Brazil* | ibrutinib  –  expansion | data from other setting,  registry | third-party payer | open system (dynamic) | annual –  4 years | current interventions | drug-only | scenario 1: fludarabine-cyclophosphamide- rituximab or ofatumumab monotherapy  scenario 2: ibrutinib monotherapy   - budget impact:   R$ 7,400,062 (year 1),  R$ 15,656,728 (year 4)  cumulative 4-year impact (per month per life insured): R$ 45,801,929  (R$0.012 to R$0.025) | not reported | not reported | simplistic  a lot of details and limitations not specifically reported |

| Parameter | Intervention | Data sources | Perspective | Target population | Time horizon | Comparator(s) | Scope of costs | Results of budget impact analysis | Sensitivity analysis | Validation | Comments and limitations |
| --- | --- | --- | --- | --- | --- | --- | --- | --- | --- | --- | --- |
| *(Pham et al., 2019),*  *USA* | glasdegib  –  combination | not reported | third-party payer | open system (dynamic) | annual –  3 years | current interventions | drug-only, condition-related | scenario 1: without glasdegib + LDAC  scenario 2: with glasdegib + LDAC   - incremental budget impact (year 1 – year 2 – year 3):   $179,290 - $262,694 - $346,098   - incremental cost PMPM (year 1 – year 2 – year 3):   $0.0149 - $0.0219 - $0.0288 | ‣ results most sensitive to the mean overall survival of glasdegib + LDAC | not reported | data sources not provided  simplistic  no limitations reported |
| *(Pribylova et al., 2016),*  *The Czech Republic* | ibrutinib  –  expansion | data from other setting | third-party payer | closed system (static) | annual –  5 years | not reported | drug-only, condition-related | scenario 1: standard of care without ibrutinib  scenario 2: standard of care with ibrutinib  national health care budget   - net budget impact year 1: 0.009% or € 1.116 million - cumulative 5-year budget impact:   0.034% or €123.786 million  oncology budget   - net budget impact in 1st year: 0.118%   cumulative 5-year budget impact: 0.368% | not reported | not reported | very simplistic  limitations not reported  no sensitivity analysis, no comparators, static population model |
| *(Pyadushkina et al., 2016), Russia* | eltrombopag  –  expansion | registry,  claims database, internal data from pharmaceutical company | third-party payer | not reported | annual –  5 years | current intervention | drug-only, condition-related | scenario 1: eltrombopag with romiplostim   - average budget savings with eltrombopag market shares increase from 20% to 50%:   €1,768,763 (year 1) and  €6,501,398 (year 5)   - cumulative 5-year budget savings: - €22,484,003 or 25.7%   scenario 2: romiplostim only | not reported | not reported | simplistic  limitations are not reported |
| *(Pyadushkina et al., 2018), Russia* | ixazomib + lenalidomide,  dexamethasone  –  expansion, substitution | claims database, market research,  registry, internal data from pharmaceutical company,  data from other setting | third-party payer | not reported | not reported –  3 years | current intervention | drug-only, condition-related | scenario 1, without ixazomib:  LD only; carfilzomib + LD  scenario 2, with ixazomib:  LD only; carfilzomib + LD; ixazomib + LD   - decrease in budgetary expenditures (with gradual increase of market share from 3% to 8%):   € 13.59 million  3-year cost savings due to lower cost of ixazomib compared to formally approved carfilzomib and its potential use in outpatient setting | not reported | not reported | additional costs excluded, simplistic  no sensitivity analysis and limitations not reported |

| Parameter | Intervention | Data sources | Perspective | Target population | Time horizon | Comparator(s) | Scope of costs | Results of budget impact analysis | Sensitivity analysis | Validation | Comments and limitations |
| --- | --- | --- | --- | --- | --- | --- | --- | --- | --- | --- | --- |
| *(Rose et al., 2017), USA* | everolimus  –  combination | published literature, claims database,  data from other setting, internal data from pharmaceutical company | third-party payer and pharmacy | closed system (static) | annual –  3 years | current interventions | drug-only, condition-related | (difference between total cost health plan of with and without everolimus)  total health plan budget impact (year 1 – year 2 - year 3)  *GI NETs*   - budget impact (per member per month):   US$0.0568 – US$0.1005 – US$0.1443   - budget impact (per year for all 1,000,000 members):   US$681,962 – US$1,206,549 – US$1,731,135  *Lung NETs*   - budget impact (per member per month):   US$0.0181 - US$0.0253 – US$0.0355  Pharmacy (year 1 – year 2 - year 3)  *GI NETs*   - budget impact (per member per month):   US$0.0606 – US$0.1072 – US$0.1538  *Lung NETs*   - budget impact (per member per month): - US$0.0204 – US$0.0285 – US$0.0396 - budget impact (per year for all 1,000,000 members):   US$216,880 – US$303,296 – US$425,995  total health plan budget impact lower than total pharmacy budget impact because of costs offsets from administration and adverse event management | ‣ variation of +/- 10% reveals most sensitive parameters as annual drug cost and treatment duration (budget impact +$2.04 cents and +$0.79 cents, respectively) | no | many assumptions influence the interpretation and credibility of results |
| *(Salazar et al., 2017), Mexico* | daratumumab  –  expansion | data from other setting | third-party payer | closed system (static) | annual –  5 years | current interventions | drug-only | percentage impact on budget of:   - NHS: 0.00062% - IMSS: 0.000408% - ISSSTE: 0.000205% - Seguro Popular: 0.002475% - SEDENA: 0.000043% - SEMAR: 0.000039% - PEMEX: 0.000006% | deterministic univariate analysis to test uncertainty of main variables, no further specifications | not reported | simplistic, no limitations reported,  only budget impact of the first year of time horizon reported |
| *(Sanon M. et al., 2012),*  *USA* | imatinib  –  substitution | published literature,  data from other setting,  internal data from pharmaceutical company | not reported | closed system (static) | annual and monthly –  3 years | current intervention | drug-only | treatment with three year versus one year of imatinib   - net budgetary impact per patient per month (year 1 – year 2):   $1090 - $2574 and 15% - 28%   - cost in PMPM: < $0.01   (in year 2 and year 3) | most sensitive to cost of imatinib and recurrence rates at year 3 | not reported | simplistic, limitations not reported |

| Parameter | Intervention | Data sources | Perspective | Target population | Time horizon | Comparator(s) | Scope of costs | Results of budget impact analysis | Sensitivity analysis | Validation | Comments and limitations |
| --- | --- | --- | --- | --- | --- | --- | --- | --- | --- | --- | --- |
| *(Savova et al., 2014),*  *Bulgaria* | nilotinib  –  expansion | registry,  internal data from pharmaceutical company,  data from other setting | patient | closed system (static) | monthly and annual –  3 years | current interventions | drug-only, condition-related | Accelerated phase   - total 6-months patient cost:   BGN 34,535.95   - total annual patient cost:   BGN 67,373.35   - drug related cost savings (year 1 – year 2 – year 3): BGN 436,359 – BGN 307,917 – BGN 307,917 - total treatment cost savings (year 1 – year 2 – year 3): BGN 441,183 – BGN 313,183 – BGN 313,183 - average 3-year drug budget cost savings: BGN 1,052,393 - total 3-year pharmaceutical budget savings: BGN 35,585   Chronic phase   - total 6-months patient cost:   BGN 33,938.90   - total annual patient cost:   BGN 66,770.90   - drug related cost savings   (year 1 – year 2 – year 3):  BGN 561,855 – BGN 307, 917 – BGN 307,917   - total treatment cost savings (year 1 – year 2 – year 3): BGN 555,222 – BGN 304,896 – BGN 304,896 - average 3-year drug budget cost savings: BGN 1,177,689 - total 3-year pharmaceutical budget savings: BGN 1,165,014 | not reported | no | simplistic  limitations not reported |
| *(Schenkel et al., 2015), USA* | ibrutinib  –  expansion | published literature, registry,  data from other setting, historical data, claims database | third-party payer | closed system (static) | annual –  1 year | current intervention | drug-only, condition-related | scenario 1: with ibrutinib   - incremental budget: $225 PTMPM - incremental budget impact per-member-per-month: $0.027 PMPM   scenario 2: without ibrutinib | ‣ model results are most sensitive to ibrutinib mean duration of therapy, followed by ibrutinib market share, and health plan population ≥ 65 years | not reported | limitations not reported, comparators not explicitly stated, very limited information,  results of sensitivity analysis not supported by numbers, short time horizon, budget impact numbers without ibrutinib were not reported |
| *(Schenkel et al., 2016),*  *USA* | ibrutinib  –  expansion | published literature, registry,  data from other setting, historical data, claims database | third-party payer | closed system (static) | annual –  1 year | current interventions | drug-only, condition-related | scenario 1: without ibrutinib   - incremental budget impact:   $4,654 PTMPM and $0.107 PMPM  scenario 2: with ibrutinib   - 1-year incremental budget impact:   $243 PTMPM, or $0.006 PMPM | ‣ model results are most sensitive to ibrutinib mean duration of therapy, followed by ibrutinib market share, and health plan population ≥ 65 years | not reported | limitations not reported, comparators not explicitly stated, very limited information  results of sensitivity analysis not supported by numbers, short time horizon |
| *(Schultz and Malone, 2014),*  *USA* | ivacaftor  –  expansion | published literature,  claims database | third-party payer | closed system (static) | annual –  not reported | not reported | drug-only | scenario 1: plan A  scenario 2: plan B  scenario 3: plan C  annual projected treatment expenditures   - health plan A: $334,476 - health plan B: $1,270,713 - health plan C: $2,631,001   premiums increase needed of $0.05 PMPM | ‣ probabilistic analysis/Monte-Carlo simulation: conduction of 1000 simulations to derive confidence intervals:  Plan A (500,000 lives): 95% CI: $306,817-$362,136  Plan B (2,000,000 lives): 95% CI: $1,169,781-$1,371,645  Plan C (4,000,000 lives): 95% CI: $2,419,785-$2,842,217 | not reported | simplistic  limitations not reported |

| Parameter | Intervention | Data sources | Perspective | Target population | Time horizon | Comparator(s) | Scope of costs | Results of budget impact analysis | Sensitivity analysis | Validation | Comments and limitations |
| --- | --- | --- | --- | --- | --- | --- | --- | --- | --- | --- | --- |
| *(Senbetta et al., 2014),*  *USA* | ibrutinib  –  combination | published literature,  historical data, claims database,  data from other setting | third-party payer | closed system (static) | monthly –  1 year | current interventions | drug-only, condition-related | scenario 1: standard of care without ibrutinib  scenario 2: standard of care with ibrutinib   - 8.3-month incremental cost: $0.010 PMPM - 12-month incremental cost: $0.027 PMPM | ‣ model results are most sensitive to ibrutinib mean duration of therapy,  followed by ibrutinib market share, and health plan population ≥ 65 years | not reported | very simplistic  limited information available  limitations not reported  very short time horizon |
| *(Serpik and Yagudina, 2015),*  *Russia* | nilotinib  –  substitution | internal data from pharmaceutical company | third-party payer | not reported | not reported –  not reported | current intervention | drug-only, condition-related | scenario 1: imatinib 1^st^ -line &  high-dose imatinib 2^nd^ -line   - annual costs per patient (1^st^ -line & 2^nd^ -line): € 6336 and € 12 672 - total expenditures: € 90 million   (1^st^ -line: € 46 million &  2^nd^ -line: € 44 million)    scenario 2: imatinib 1^st^ -line &  nilotinib 2^nd^ -line   - annual costs per patient (1^st^ -line & 2^nd^ -line): € 6336 and € 35 040 - total expenditures: € 82,2 million   including nilotinib leads to a budget increase but does not exceed total current (2014) expenditures for CML | not reported | not reported | simplistic  no sensitivity analysis and limitations not reported  limited data source reporting |
| *(Soto Molina et al., 2017),*  *Mexico* | brentuximab vedotin  –  expansion | published literature,  data from other setting | third-party payer | open system (dynamic) | annual –  5 years | current intervention | drug-only | scenario 1: with brentuximab vedotin   - incremental investment (2016 and 2020):   $169,322 and $893,529   - annual average investment (2016):   $526,914 or 0.016% of NHS budget  scenario 2: without brentuximab vedotin | not reported | not reported | simplistic  only accounted for influx of new patients and not for efflux of patients |
| *(Soto Molina et al., 2018),*  *Mexico* | bosentan  –  expansion | data from other setting, expert opinion | third-party payer | not reported | annual –  5 years | current intervention | drug-only | scenario 1: support and specific therapy  scenario 2: bosentan + support and specific therapy   - budget impact year 1:   US$ 318,064.56  (0.008251% of NHS budget)   - budget impact year 5:   US$ 1,576,135.12  (0.04215% of NHS budget)   - average 5-year investment:   US$ 948,449.78  (0.02536% of NHS budget) | not reported | not reported | simplistic  many details not reported |
| *(Stellato et al., 2018),*  *USA* | dabrafenib & trametinib  –  expansion | claims database, registry,  expert opinion, internal data from pharmaceutical company,  data from other setting | third-party payer | closed system (static) | monthly –  3 years | current interventions | drug-only, condition-related | reference scenario without dabrafenib and trametinib:   - cumulative healthcare costs: $6.3 million - cumulative 3-year budget impact:   $549 thousand or 1.5 cents PMPM  new scenario with dabrafenib and trametinib:  cumulative healthcare costs: $6.9 million | not reported | Yes (indirect treatment comparison with other randomized control trials) | no sensitivity analysis  no yearly numbers reported on budget impact only totals  static population model  no limitations reported  simplistic |

| Parameter | Intervention | Data sources | Perspective | Target population | Time horizon | Comparator(s) | Scope of costs | Results of budget impact analysis | Sensitivity analysis | Validation | Comments and limitations |
| --- | --- | --- | --- | --- | --- | --- | --- | --- | --- | --- | --- |
| *(Tafazzoli et al., 2018),*  *USA* | moxetumomab  –  expansion | expert opinion, internal data from pharmaceutical company, data from other setting | third-party payer | closed system (static) | monthly –  1 year | current interventions | drug-only, condition-related | with moxetumomab  incremental cost: $0.01 PMPM | findings were robust in sensitivity analyses with most sensitive inputs being moxetumomab market uptake and percentage of eligible patients seeking treatment | not reported | simplistic  budget impact not calculated with and without orphan drug  short time horizon, no validation,  static population model  no limitations reported, data sources not mentioned per specific parameter |
| *(Tremblay et al., 2019),*  *USA* | eltrombopag  –  combination | internal data from pharmaceutical company,  published literature,  data from other setting, claims database | third-party payer | open system (dynamic) | annual and monthly –  3 years | current intervention | drug-only, condition-related | scenario 1: IST without eltrombopag  scenario 2: IST with eltrombopag   - absolute budget impact (baseline - year 1 - year 2 - year 3 – total):   $0 – $8,257,266 – $16,631,858 – $25,125,028 – $50,014,152   - relative budget impact (baseline - year 1 - year 2 - year 3 – total):   0% - 8% - 17% - 25% - 17%  per-person-per-month budget impact (year 1 – year 2 – year 3 – total): $2.08x10^−9^ – $4.17x10^−9^ – 6.25x10^−9^ – $4.18x10^−9^ | ‣ variation of +/- 20% greatest impact on cost of eltrombopag, incidence of severe aplastic anemia and complete response rates while on eltrombopag | not reported | market shares based on manufacturer's internal estimates so limited accuracy  limited research on incidence and prevalence, indirect costs not analysed  not accounted for Medicare and other discounts thus overestimation of budget impact  no incorporation of real-world data because model used clinical trial data, short trial length duration  long-term treatment not predictable, mortality data were modelled and not collected  only accounted for increasing market shares of the treatment and not for decrease market share due to efflux of patients |
| *(Tritaki et al., 2016),*  *Greece* | nintedanib  –  expansion | internal data from pharmaceutical company, expert opinion,  data from other setting | third-party payer | not reported | annual –  5 years | current intervention | drug-only, condition-related | scenario 1: budget impact of pirfenidone and best supportive care (current)  scenario 2: budget impact of nintedanib   - cost of exacerbation events compared to current situation (2016 – 2020): €4,154 - €15,385 - incremental budget impact (2016): €2,088,281   cost savings (2016 – 2020): 0.10% - 0.20% due to avoided acute exacerbations | not reported | yes  (Greek key opinion leader) | simplistic  limited information available  no information on population or sensitivity analysis  no limitations reported |
| *(Truong et al., 2014),*  *USA* | pasireotide  –  expansion | published literature,  claims database, registry,  expert opinion | third-party payer and pharmacy | open system (dynamic),  closed system (static) | monthly and annual –  3 years | current interventions, off-label use of interventions | drug-only, condition-related | scenario 1: without pasireotide  scenario 2: with pasireotide  entire health plan/total budget impact   - budget impact in US$ (year 1 – year 2 – year 3): 0.0115 PMPM - 0.0184 PMPM - 0.0194 PMPM - budget impact in US$ (per year for all 1,000,000 members) (year1 – year 2 – year 3): US$137,505 – US$219,892 – US$231,954   pharmacy   - budget impact in US$ (year 1 – year 2 – year 3): 0.0257 PMPM –   0.0363 PMPM - 0.0360 PMPM | ‣ variation of +/- 10% → budget impact +$0.01  whereas variation of +/- 25% → budget impact +$0.015  most sensitive parameters are pasireotide response and clinical benefit rate | no | simplistic  limitations are not mentioned |

| Parameter | Intervention | Data sources | Perspective | Target population | Time horizon | Comparator(s) | Scope of costs | Results of budget impact analysis | Sensitivity analysis | Validation | Comments and limitations |
| --- | --- | --- | --- | --- | --- | --- | --- | --- | --- | --- | --- |
| *(Venturini et al., 2008),*  *Italy* | deferasirox  –  substitution | registry,  data from other setting, claims database | third-party payer | closed system (static) | annual –  not reported | current interventions | drug-only, condition-related | - estimated annual costs deferoxamine:   €734,808 - €1,327,797   - estimated annual costs deferasirox:   €3,615,382 - €5,575,618  scenario 1: from deferoxamine to deferasirox   - expenditure increase per year:   €2,881,074 - €4,247,823  scenario 2: switch from deferoxamine to deferiprone   - expenditure increase per year:   €2,858,547 - €4,283,481 | not reported | not reported | alternative scenarios should be considered  (patients switching to new drug only or patients whose quality of life is strongly affected by the infusion pump) |
| *(Villa et al., 2017),*  *Italy* | idelvion  –  expansion | registry, historical data,  internal data from pharmaceutical company | third-party payer | closed system (static) | not reported –  3 years | current interventions | drug-only | Idelvion   - net 3-year budget savings:   €7.5 million  regional differences in budget impact with   - Lombardy: €1.3 million   Trentino Alto-Adige: €50.000 | most sensitive to drug dosages | not reported | limitations: conservative assumptions, positive clinical and economic effects of haemorrhagic complications reductions were not considered |
| *(Wehler et al., 2015a),*  *Mexico* | bortezomib subcutaneous  –  substitution | expert opinion,  published literature,  internal data from pharmaceutical company | third-party payer | not reported | annual –  not reported | current intervention | drug-only, condition-related | bortezomib intravenous   - annual per patient treatment costs: $653,136   (drug: $495,486, administration: $148,864 and adverse events: $8,786)  bortezomib subcutaneous   - annual per patient treatment costs: $532,877   (drug: $495,513, administration: $30,906 and adverse events: $6,458) | not reported | not reported | simplistic  limitations not reported |
| *(Wehler et al., 2015b),*  *Venezuela* | generic bortezomib intravenous  –  expansion | expert opinion,  published literature,  internal data from pharmaceutical company | third-party payer | closed system (static) | annual –  not reported | current interventions | drug-only, condition-related | current market share   - treatment costs: $27,699,084 or $102,510/patient   market with generic bortezomib   - treatment costs: $34,944,197 or $129,323/patient or 26.2% increase (assuming market share of 20%)     increase is driven by increased utilization of intravenous bortezomib ($20,155/patient) and administration costs ($4,562/patient) and adverse events costs ($2,096/patient) | not reported | not reported | another option to promote savings is using newer treatment alternatives that provide high efficacy and favourable adverse events and administration profiles |
| *(Weidlich et al., 2018),*  *United Kingdom* | isavuconazole  –  substitution | not reported | third-party payer | closed system (static) | annual –  5 years | current intervention | drug-only, condition-related | scenario 1: current (voriconazole) without isavuconazole  scenario 2: future scenario (voriconazole) with isavuconazole   - total 5-year spending: increase of 2.1% or £98,701,886 or £28,699 per patient - overall annual spending: average 2.1% increase   small increase in total spending due to several cost offsets | not reported | not reported | cost offsets by reduction of hospitalisations, adverse events, laboratory analysis and deaths  no sensitivity analysis  limitations not reported  limited information on data sources |
| *(Whalen et al., 2013),*  *United Kingdom* | everolimus  –  expansion | internal data from pharmaceutical company,  data from other setting | third-party payer | open system (dynamic) | annual –  5 years | current intervention | drug-only, condition-related | with everolimus   - average treatment cost: £4,600,000   (year 1: £2,700,000; year 5: £6,200,000)   - incremental 5-year spending:   decreased with £54,000  annual per patient treatment cost: £31,000 | most sensitive to:  patient prevalence, percent eligible for everolimus, and percent experiencing ≥ 30% AML volume reduction | not reported | discounted at 3.5% per annum  simplistic  no limitations reported  not specifically ‘with and without treatment’  calculations not depicted |

| Parameter | Intervention | Data sources | Perspective | Target population | Time horizon | Comparator(s) | Scope of costs | Results of budget impact analysis | Sensitivity analysis | Validation | Comments and limitations |
| --- | --- | --- | --- | --- | --- | --- | --- | --- | --- | --- | --- |
| *(Xu et al., 2017), China* | nilotinib  –  substitution | published literature, expert opinion,  internal data from pharmaceutical company | third-party payer | open system (dynamic) | annual – 5 years | current intervention | drug-only | scenario 1: imatinib as first line treatment (current)  scenario 2: nilotinib as first line treatment  total budget impact of 5 years: 1.99% decrease after introduction of nilotinib compared to current scenario | not reported | not reported | discount rate of 3%  simplistic  limitations not reported |
| *(Yagudina et al., 2015a), Russia* | high purity concentrate VWF:Rco/  FVII=0,9/1  –  substitution | published literature, expert opinion,  registry | not reported | not reported | annual –  1 years | current interventions | drug-only, condition-related, indirect | total cost reduction per all population:  scenario 1: switch from no prophylaxis to  prophylaxis 3: 21906000 rubles ($ 396 360)  scenario 2: switch from prophylaxis 1 to prophylaxis 3: 22143000 rubles ($ 400 648)  scenario 3: switch from prophylaxis 2 to prophylaxis 3: 5752000 rubles ($104 075) | not reported | not reported | simplistic  no sensitivity analysis and limitations not reported |
| *(Yagudina et al., 2015b), Russia* | canacinumab  –  substitution | expert opinion | not reported | closed system (static) | annual –  1 years | current intervention | drug-only, condition-related | canacinumab treatment group vs.  retrospective group with only symptomatic treatment   - cost of canacinumab:   40,830,937 RUB or € 668,220   - difference in required budget funds canacinumab and symptomatic therapy: 38,787,076 RUB or € 634,771 - decrease direct medical costs canakinumab: 763,705 RUB or € 12,498 | not reported | not reported | canacinumab leads to budget spending but reduces direct medical costs, simplistic  no sensitivity analysis and limitations not reported |
| *(Yang et al., 2018),*  *USA* | tisagenlecleucel  –  expansion | market research,  historical data,  claims database,  registry, published literature | third-party payer | closed system (static) | annual and monthly –  5 years | current interventions | drug-only, condition-related | scenario 1: treatment with tisagenlecleucel   - incremental total budget impact: $240,125 (year 1) - $689,779 (year 2) – $841,686 (year 3) –   $764,462 (year 4) - $742,099 (year 5)   - incremental PMPM: $0.02 (year 1) - $0.06 (year 2) –   $0.07 (year 3) - $0.06 (year 4) – $0.06 (year 5)  scenario 2: treatment without tisagenlecleucel | not reported | not reported | limitations not reported  concise analysis |
| *(Zhang et al., 2016),*  *USA* | pasireotide LAR  –  expansion | published literature, claims database,  internal data from pharmaceutical company,  data from other setting, registry,  market research, expert opinion | third-party payer and pharmacy | open system (dynamic) | annual –  3 years | current interventions | entire health plan:  drug-only, condition-related  –  pharmacy budget: drug-only | scenario 1: health plan formulary with pasireotide LAR  managed care health plan   - total difference in budget impact   *1^st^ line* (year 1 - year 2 - year 3):  $36,864/$0.0031PMPM - $93,079/$0.0078PMPM - $169,986/$0.0142PMPM  *2^nd^ line* (year 1 - year 2 - year 3):  $27,792/$0.0023PMPM - $69,432/$0.0058PMPM - $142,250/$0.0119PMPM  pharmacy budget   - total difference in budget impact   *1^st^ line* (year 1 - year 2 - year 3):  $0.0031PMPM - $0.0077PMPM - $0.0141PMPM  *2^nd^ line* (year 1 - year 2 - year 3):  $0.0023PMPM - $0.0057PMPM - $0.0117PMPM  scenario 2: health plan formulary without pasireotide LAR  pasireotide LAR causes a very small increase in costs of the health plan | ‣ varying 5 parameters with  +/- 10% →  most significant impact on pasireotide's drug price +/- 0.50 cents PMPM over 3 years | not reported | perspectives of US managed care health plan (entire health plan) and pharmacy budget  limited data sources on eligible patient population and market shares  published clinical and economic data is limited  comorbidity costs of general population were used which may likely be an underestimation of costs offsets due to controlled disease and an overestimation of overall budget impact of pasireotide LAR |

*with ten studies published before ISPOR budget impact analysis good practice principles in *(Sullivan et al., 2014)*

(*AML*, angiomyolipoma. *CML*, chronic myelogenous/myeloid leukemia. *CS*, carcinoid syndrome. *GI*, gastro-intestinal. *HAEI/II*, hereditary angioedema I/II. *IPF*, idiopathic pulmonary fibrosis. *IST*, immunosuppressive therapy. *IU*, international unit. *LD*: lenalidomide and dexamethasone. *LDAC*, low-dose cytarabine. ‘Mexican public institutions’: *IMSS*, Instituto Mexicano del Seguro Social. *ISSSTE*, Instituto de Seguridad y Servicios Sociales de los Trabajadores del Estado. *PEMEX*, Petróleos Mexicanos. *SEDENA*, Secretaría de Defensa Nacional. *SEMAR*, Secretaría de Marina. *NETs*, Neurendocrine Tumors. *NHS*, national health service. *PMPM*, per member per month. *PTMPM*, per treated member per month. *uHCC*, unresectable hepatocellular carcinoma. *VWF:Rco/FVII*, Von Willebrand Ristocetin Cofactor/Factor VII)
