# Supplementary material for: Methodological Quality Assessment of Budget Impact Analyses for Orphan Drugs: A Systematic Review
Source: Front Pharmacol. 2021 Apr 21;12:630949. doi: 10.3389/fphar.2021.630949 (PMC8098807; doi:10.3389/fphar.2021.630949)
Supplement: Supplementary file 2 [file table2.docx]

**Supplementary Table 2.** Characteristics of budget impact analyses for combination of orphan drugs*

| **Parameter** | **Number of orphan drugs** | **Data sources** | **Perspective** | **Target population** | **Time horizon** | **Scope of costs** | **Results of budget impact analysis** | **Sensitivity analysis** | **Validation** | **Comments and limitations** |
| --- | --- | --- | --- | --- | --- | --- | --- | --- | --- | --- |
| *(Alonso Martinez et al., 2018),*  *Spain* | 51 | registry,  data from other setting | hospital, pharmacy | closed system (static) | annual – 1 year | drug-only | *retrospective (2016)*  hospital outpatient paediatric pharmacy   - % of total pharmaceutical expenditure: 36.3% or €1,379,400 of €3.8million - drugs for rare disease treatment accounted for a total of: €7.7 million   paediatric day hospital   - % of total pharmaceutical expenditure: 71% or €2,769,000 of €3.9 million | not reported | not reported | orphan drugs had the greatest economic impact on both hospital and pharmacy budget  very limited information, short time horizon,  no assumptions reported |
| *(Denis et al., 2010a), Belgium* | 30 (2008)  –  not reported for 2018-2013 | published literature, claims database,  registry,  data from other setting | hospital | not applicable | annual – 5 years | drug-only | *retrospective (2008)*   - total budget impact: €66.2 million of which   5% hospital drug budget, 1.6% hospital budget, 1.9% pharmaceutical expenditure and  0.3% Belgium’s health expenditure  *prospective (2008-2013)*   - total budget impact (scenario 2): €162 million - increase of budget impact: 145% - all drug reimbursement costs: 4% - hospital drug budget: 10% | scenario:  scenario 1: low-growth (budget impact: €130 million and costs: €2.0 million/drug/year)  scenario 2: medium-growth (budget impact: €162 million and costs: €2.135 million/drug/year)  scenario 3: high-growth (budget impact: €204 million and costs: €2.3 million/drug/year) | Yes  (cross-checking data with multiple data sources) | considered three scenarios  one orphan excluded (thalidomide)  number of patients is unknown  Belgian Special Solidarity Fund reimbursement not included  did not consider market exit of orphan drugs |
| *(Divino et al., 2016a),*  *USA* | 316 | claims database,  market research,  historical data,  registry,  internal data from pharmaceutical company, published literature | third-party payer | not applicable | annual –  12 years | drug-only | *retrospective (2007-2013)*   - total orphan drug expenditure (in 2014 USD, Billions): 15.030 - 30.048 - total pharmaceutical expenditure (in 2014 USD, Billions): 311.384 - 337.442 - % of total orphan/pharmaceutical drug expenditure: 4.8 - 8.9   *prospective (2014-2018)*   - total orphan drug expenditure (2014 USD, Billions): 33.5 - 44.2 - total pharmaceutical expenditure (in 2014 USD, Billions): 380.8 - 465.0 or 22.1% increase - % of total orphan/pharmaceutical drug expenditure: 8.8% - 9.5%   orphan drug expenditure as a percentage of total pharmaceutical expenditures has remained fairly stable | not reported | yes (historical spending trends, comparison with former Europe budget impact analyses) | no stratification of results by therapies (chronic vs. acute condition) might have long term impact on payer's budget  possible underestimation of orphan drug sales due to limited MIDAS database coverage  extrapolation of historical data from 2007-2013 for future linear trend analysis of 2014-2018  not accounted for changing trends may result in underestimation of forecasted orphan drug spending  orphan drugs approved by US/FDA used as proxy for orphan drug landscape in Canada, additional approved indications in Canada that are not approved in the USA might have been missed  not specifically accounted for off-label orphan drug use  focused on brand orphan drugs only and did not include generics |

| **Parameter** | | **Number of orphan drugs** | **Data sources** | **Perspective** | **Target population** | **Time horizon** | **Scope of costs** | **Results of budget impact analysis** | **Sensitivity analysis** | | **Validation** | **Comments and limitations** |
| --- | --- | --- | --- | --- | --- | --- | --- | --- | --- | --- | --- | --- |
| *(Divino et al., 2016b), Canada* | 147 | | claims database,  market research,  historical data,  registry,  internal data from pharmaceutical company, published literature | not reported | not applicable | annual –  12 years | drug-only | *retrospective (2007-2013)*   - total orphan drug expenditure (2014 CAD, millions): 610.2 - 1100.0 - total pharmaceutical expenditure (in 2014 CAD, millions): 18,233.6 -19,665.7 - % of total orphan/pharmaceutical drug expenditure: 3.3 - 5.6   *prospective (2014-2018)*   - total orphan drug expenditure (2014 CAD, millions): 1,151.2 - 1,472.2 or 27.9% increase - total pharmaceutical expenditure (in 2014 CAD, millions): 20,413 - 25,255 or   23.7% total orphan drug increase   - % of total orphan/pharmaceutical drug expenditure: 5.6 - 5.8   orphan drug expenditure will remain fairly low and stable below 6% of total pharmaceutical expenditure | not reported | | Yes (historical spending trends, statistical validation, comparison with USA and Europe budget impact analyses) | possible underestimation of orphan drug sales due to limited MIDAS database coverage  extrapolation of historical data from 2007-2013 for future linear trend analysis of 2014-2018  not accounted for changing trends may result in underestimation of forecasted orphan drug spending  orphan drugs approved by US/FDA used as proxy for orphan drug landscape in Canada, additional approved indications in Canada that are not approved in the USA might have been missed  not specifically accounted for off-label orphan drug use    focused on brand orphan drugs only and did not include generics |
| *(Flostr et al., 2016), Europe* | 99 | | registry,  historical data | not reported | not applicable | annual – 10 years | drug-only | *prospective (2016-2025)*   - total budget impact: €5.1Bn in 2015 to €22Bn or   14% of total drug spend    → healthcare budgets will be under significant pressure due to rising orphan drug expenditure | | not reported | yes (IMS MIDAS database for input values) | very concise analysis  rising prices are not a major budget growth driver  no detail of mathematical conversion rates and limitations not reported |
| *(Fontanet et al., 2018), Spain* | 83 | | claims database | third-party payer | open system (dynamic) | annual – 5 years | drug-only | *retrospective (2013-2017)*   - treatment cost (2013-2017): € 84M - € 157M - % of total pharmaceutical expenditure (2013-2017): 4.3 - 6.7   sustainable access to orphan drugs should be guaranteed through new measures because of growing expenditure | | not reported | yes (published data in comparable environments) | no sensitivity analysis  very concise information  no limitations reported |

| **Parameter** | **Number of orphan drugs** | **Data sources** | **Perspective** | **Time horizon** | **Scope of costs** | **Results of budget impact analysis** | **Sensitivity analysis** | **Validation** | **Comments and limitations** |
| --- | --- | --- | --- | --- | --- | --- | --- | --- | --- |
| *(Forte et al., 2019),*  *Canada* | 32 | registry,  published literature,  claims database,  data from other setting | third-party payer | annual –  9 year | drug-only | *retrospective (2010-2018)*   - total public drug plan costs (2010 - 2014 – 2018):   $14.8 million - $78.7 million – $182.8 million   - orphan drug expenditure as a proportion of total public drug expenditure (2014 – 2018): 0.7% to 1.3% - total public drug plan costs (2014 – 2018):   $11.4 billion to $14.4 billion  ultra-orphan drugs (~40% of total orphan drugs) contributed ~60% of total orphan drug expenditure; future extrapolated costs showed similar trend in growth  cost of orphan drugs in Canada is projected to remain a small fraction of total public drug expenditures | not reported | not reported | no annual numbers on budget impact  no numbers on extrapolation for future costs  used EMA as a reference for orphan drugs  no limitations reported |
| *(Gea et al., 2013),*  *Andorra* | 11 | registry,  claims database,  data from other setting | hospital | annual –  1 year | drug-only | *retrospective (2011-2012)*  budget impact of pharmaceutical hospital budget:  8.2% (16.5% of this percentage refers to off-label orphan drug use) | not reported | not reported | very concise, a lot of information missing  limitations and assumptions not reported  budget impact not supported by elaborate numbers |
| *(Hajimiri et al., 2019),*  *Iran* | 42 | registry,  claims database | third-party payer | annual –  1 year | drug-only | *retrospective (2018)*   - total cost of orphan drugs: US$ 160.95 million - total coverage of products by national health insurance:   US$113.6 million or 71%   - growth rate since 2017: 1.9% - % of total pharmaceutical expenditure in Iran: 3.2 - estimated growth rate to 2024: 12%   bevacizumab, infliximab, antihemophilic factor VII accounted for 55% of total orphan drug expenditure | not reported | not reported | limitations not reported  concise  assumptions on budget impact numbers not reported |
| *(Heemstra et al., 2010),*  *Denmark/*  *France/*  *Belgium* | 41 (Denmark)  34 (France)  not reported (Belgium) | data from other setting | not reported | annual –  5 years | drug-only | *retrospective (2005-2009)*  Denmark   - Total budget impact:   €20.3M (2005) - €58.3M (2009)   - proportion of outpatient drugs of total BI: 0.01% (2005) - 1.55% (2009)   France 2002 2008   - Total budget impact:   €71M (2002) - €496M (2008)   - proportion total sold drugs (2008):   1.8%  Belgium   - Total budget impact (2008): €66.2M - proportion of total reimbursed drug costs (2008): 2%   total budget impact is modest although cost per patient is relatively high | not reported | yes (comparison with budget impact analyses of France and Belgium) | no sensitivity analysis  lack of quantitative and more specific data for France and Belgium  limitations not reported |

| **Parameter** | **Number of orphan drugs** | **Data sources** | **Perspective** | **Time horizon** | **Scope of costs** | **Results of budget impact analysis** | **Sensitivity analysis** | **Validation** | **Comments and limitations** |
| --- | --- | --- | --- | --- | --- | --- | --- | --- | --- |
| *(Hutchings et al.),*  *Sweden/*  *France* | 152 | historical data,  claims database | not reported | annual – 21 years | drug-only | Sweden  *retrospective (2000-2012)*   - average sales growth: SEK 11.1 million (year 4 post-launch), SEK 21.5 million (year 12 post-launch) - average annual market growth rate (2000-2012):   SEK 20.3 – 36.8 billion or 4%   - Budget impact growth rate (% of total pharmaceutical expenditure): 2.5 (2012)   *prospective (2013-2020)*   - Budget impact growth rate (% of total pharmaceutical expenditure): 2.7 (2013) - 3.9 (2018) - 4.1 (2020): plateau   France  *retrospective (2000-2012)*   - average sales growth: €13.4 million (year 4 post-launch), €21.6 million (year 12 post-launch) - average annual market growth rate (2000-2012):   € 20.5 – 34.0 billion or 3%   - budget impact growth rate (% of total pharmaceutical expenditure): 3.1 (2012)   *prospective (2013-2020)*   - budget impact growth rate (% of total pharmaceutical expenditure): 3.2 (2013) - 4.6 (2018) - 4.9 (2020): plateau   budget impact is relatively small proportion of  total pharmaceutical expenditure | budget impact variation of 2% - 11% in peak year  most sensitive parameters: success rate for designated orphan drugs obtaining market authorization approval and average total annual sales post-launch | yes (identical analysis on USA orphan drug data, comparison with former Europe budget impact analyses) | accounted for price adjustments following loss of intellectual property protections  limitations: dynamic forecasting model has potential weaknesses such as assumptions, impossible to have precision in predictions |
| *(Kanters et al., 2014),*  *Netherlands* | total of 32:  11 (2006)  22 (2007)  26 (2008)  31(2009)  36 (2010)  41 (2011)  43 (2012) | claims database, published literature, data from other setting,  registry,  author assumptions | not reported | annual –  6-years | drug-only | *retrospective (2006-2012)*   - total pharmaceutical expenditure: 1.1% (2006) to 4.2% (2012) is an increase with 12.6% or quadrupled - growth rates: 60.1% (2006-2007) to 7.9% (2011-2012), decreased over time - average annual treatment costs (2012): € 255.615 (inpatient drugs) and € 40.679 (outpatient drugs)   price changes were modest with slight decrease (-1.2%)     - budget impact: €61.2 million - €260.4 million   ‣ 326% increase in absolute terms  ‣ 278% increase relative to total pharmaceutical expenditure | most sensitive parameters are inpatient drugs with unavailable data and extrapolation | yes (comparison with budget impact analyses of other European countries) | accounted for inpatient and outpatient drugs  limitations: limited time period, longer study period needed to investigate future trends in growth rate and budget impact and more detailed information, not accounted for generics |

| **Parameter** | **Number of orphan drugs** | **Data sources** | **Perspective** | **Time horizon** | **Scope of costs** | **Results of budget impact analysis** | **Sensitivity analysis** | **Validation** | **Comments and limitations** |
| --- | --- | --- | --- | --- | --- | --- | --- | --- | --- |
| *(Kim and Oh, 2017),*  *Korea* | 91 (2010) +  117 (2015) | registry,  claims database | not reported | annual – 6 years | drug-only | *retrospective (2010-2015) and prospective (2016-2020)*   - orphan drug expenditure:   86 billion KRW or 73 million USD (2010) and 229 billion KRW or 195 million USD (2015)   - % of total Korean pharmaceutical expenditure: 0.687% (2010) and 1.646% (2015) - 1.9% (2016) and 3.4% (2020) - CAGR: 17.7% for orphan drugs and 1.8% for total Korean pharmaceutical expenditure   budget impact is very small compared to total pharmaceutical expenditure and increasing very fast annually | not reported | not reported | rare disease in Korea is disease affecting 1 in 20,000 persons  no sensitivity analysis  no validation  no limitations reported  budget impact calculations not transparently stated  limited information |
| *(Klimes et al., 2012),*  *The Czech Republic* | 49 | registry,  claims database | not reported | annual – 7 years | drug-only | *retrospective (2004-2010)*   - expenditures (EUR, million) (2004 - 2010):   0.2, 2.5, 12.8, 31.9, 51.2, 66.3 and 83.4   - % of total pharmaceutical expenditures (2006-2010):   0.8 (2006), 1.8 (2007), 2.3 (2008), 3.0 (2009),  3.6 (2010)   - current orphan drug expenditure:   4% of total drugs expenditures  pharmaceutical expenditure is comparable to other EU countries | not reported | yes (comparison to expenditures of EU countries) | drug-only costs, very simplistic  budget impact only reported for 2006-2010  concise, no sensitivity analysis  conditional or exceptional reimbursement not considered |
| *(Li et al., 2018),*  *USA* | 5 | registry,  claims database, internal data from pharmaceutical company,  published literature | pharmacy | monthly – 1 year | drug-only | *prospective*   - PMPM from $0.014 to $0.272:   deutetrabenazine: $0.049, amantadine hydrochloride: $0.146, tasimelteon: $0.272, dichlorphenamide: $0.014,  obeticholic acid: $0.051 | PMPM range could be much greater ($0.007 to $0.409)  most sensitive parameters are prevalence and patient dosing | yes (input values compared to company sales and database) | comparative cost determination framework used  short time horizon  year of analysis not explicitly stated  no limitations reported |

| **Parameter** | **Number of orphan drugs** | **Data sources** | **Perspective** | **Target population** | **Time horizon** | **Scope of costs** | **Results of budget impact analysis** | **Sensitivity analysis** | **Validation** | **Comments and limitations** |
| --- | --- | --- | --- | --- | --- | --- | --- | --- | --- | --- |
| *(Logviss et al., 2016),*  *Latvia* | 21 | claims database,  registry,  published literature | third-party payer | not applicable | annual – 5 years | drug-only | *retrospective (2010-2014)*   - annual per patient expenditure:   averaged at € 23 701 and  varied between € 1 534 - € 580 952   - orphan drug expenditure: €2 065M - €3 065M (annual) – total over 5 years: €12 467M - % of total pharmaceutical market (2014):   0.84% with max 1.04% (2012) and min 0.70% (2013)   - % of total drug reimbursement budget: 2.14% with max 2.62% (2012) and min 1.83% (2013)   budget impact is extremely small compared to  West -European countries, it will grow and remain sustainable and relatively small  current reimbursement budget is insufficient for most orphan drugs | not reported | yes (comparison with budget impact analyses of other European countries) | oncology represented biggest percentage (52.99%) of orphan drug expenditure  accounted for generics influence on budget impact  limitations:  only one third- party payer’s perspective,  actual drug costs may be higher,  different approaches for estimating the number of patients receiving particular drugs (national reports, only considered daily doses used for main indications in adults),  only expenditures related to orphan indication were considered |
| *(Morginstin et al., 2019), Israel* | 41 | registry | third-party payer | not applicable | not reported – 17 years | drug-only | *retrospective (2002-2017)*   - total 17-year orphan drug expenditure:   316 million NIS or  4.4% of entire Health basket budget  unsustainable cost escalation is currently not justified | not reported | not reported | only one net total value budget impact reported  concise, no sensitivity analysis  longitudinal study without any details  not specified which orphan drugs reimbursed  data sources very limited |
| *(Ramos Santana et al., 2018),*  *Spain* | 4 | not reported | hospital | closed system (static) | monthly and annual – 1 year | drug-only | - total cost of medications: €199,029.94 (monthly) and €2,388,359.24 (yearly)   orphan drug budget impact is 4% of hospital's budget | not reported | not reported | very concise analysis, limitations not reported,  orphan disease used as proxy for orphan drug, budget impact not supported by hard numbers  no sensitivity analysis  no validation |

| **Parameter** | **Number of orphan drugs** | **Data sources** | **Perspective** | **Time horizon** | **Scope of costs** | **Results of budget impact analysis** | **Sensitivity analysis** | **Validation** | **Comments and limitations** |
| --- | --- | --- | --- | --- | --- | --- | --- | --- | --- |
| *(Schey et al., 2011),*  *Europe* | not reported but predicted 5 new orphan diseases per year | historical data,  data from other setting,  registry,  internal data from pharmaceutical company,  published literature | not reported | annual – 20 years | drug-only | *prospective 2010-2020*   - annual per patient cost: €1,251 - €407,631 and median €32,242 - orphan drug cost: €5M/year in 2002, €143M/year in 2010, €110M/year in 2020 (steady-state) - total pharmaceutical market share: increase from 3.3% in 2010, peak of 4.6% in 2016, between 4% and 5% in 2020 (steady-state)   budget impact is likely to plateau between 4% and 5% in terms of growth in cost as a proportion of total pharmaceutical expenditure | ‣ variation of parameters: budget impact within 3% - 6.6% of all total pharmaceutical market sales  ‣ structural uncertainty: use of orphan  diseases as unit of measurement instead of orphan drugs  ‣ most sensitive parameters: % of products with orphan designation that achieve market authorisation followed by average cost of orphan disease per year and Eurozone + UK pharmaceutical market value in 2010 | yes (comparison with budget impact analyses of other European countries) | primary unit of measurement is orphan disease instead of orphan drug |
| *(Schlander et al., 2015),*  *Europe* | 18 (approved ultra-orphan drugs) + 29 (ultra-orphan drugs in the pipeline) | published literature assumptions,  internal data from pharmaceutical company,  data from other setting,  registry,  data from other setting | third-party payer | annual – 10 years | drug-only | *prospective (2012-2021)*   - total budget impact over 10 years: €20,625 million, €15,660 million for approved ultra-orphan drugs and €4965 million for pipeline ultra-orphan drugs - % of pharmaceutical expenditure in 2021: 1.4 - % increase of budget impact over 10 years: 169 - total 5-year budget impact:   €7483 million  no concerns for uncontrolled growth in expenditures but budget impact should continuously be monitored | most sensitive parameters are   - market penetration rate:   if 10% → budget impact €11,018 million (0.8% of total pharmaceutical expenditure)  if 30% → budget impact is €25,728 (1.8% of total pharmaceutical expenditure)   - annual growth rate in sales volume:   if 5% → budget impact is €17,430 million (1.2% of total pharmaceutical expenditure)  if 15% → budget impact is €24,904 (1.7% of total pharmaceutical expenditure) | yes – (comparison with *(Schey et al., 2011)* budget impact study) | 3.5% discount rate  drugs for ultra-orphan diseases used and not necessarily 'orphan drugs' thus overestimation of sales of ultra-orphan drugs  uncertainty about duration of market exclusivity  assumed fixed growth rate is not necessarily right  sales data used as an approximation of payers' expenditures  lack of data about avoidance of clinical events and reductions in morbidity so not accounted for potential savings |
| *(Sujkowska et al., 2015), Poland* | 28 | registry | third-party payer | annual – 2 years | drug-only | *retrospective*   - total public payer reimbursement spending (2012-2013): €2.41 billion - €2.26 billion   orphan drug percentage of total healthcare budget or budget impact (2012-2013): 1.5% - 3.2% | not reported | yes (official website of EMA compared to official reimbursement list of the Ministry of Health to determine which orphan drugs to include) | very concise  limited information about budget impact numbers and calculations  limitations not reported  short time horizon  limited data sources reported |

*with five studies published before ISPOR budget impact analysis good practice principles in *(Sullivan et al., 2014)*

(*CAGR*, compound annual growth rate. *EMA*, European Medicines Agency)
