# Supplementary material for: Methodological Quality Assessment of Budget Impact Analyses for Orphan Drugs: A Systematic Review
Source: Front Pharmacol. 2021 Apr 21;12:630949. doi: 10.3389/fphar.2021.630949 (PMC8098807; doi:10.3389/fphar.2021.630949)
Supplement: Supplementary file 3 [file datasheet1.docx]

Supplementary Material

# Protocol

1. ***Review question***

What is the methodological quality of budget impact analyses of orphan drugs according to ISPOR good practice guidelines?

1. ***Searches***

Online databases of PubMed, Embase and Value in Health will be searched for “budget impact analysis” and “orphan drugs”. An additional manual search of Google will be performed to capture possibly missed articles.

1. ***PICO (participants, interventions, comparators, outcomes)***

*Participants:* Studies in publicly available literature reporting a budget impact analysis of one individual orphan drug or a collection of orphan drugs in a healthcare system.

*Interventions:* Medicines for the treatment of rare diseases that are listed as orphan drugs by the US FDA and/or listed as orphan medicinal products by EMA, for the indication described in the study.

*Comparators:* Standard treatment, namely ISPOR good practice guidelines for budget impact analyses (2017 and 2014).

*Outcomes:* methodological quality assessment and recommendations for future budget impact analyses based on the considered ISPOR characteristics: 1) Perspective 2) Target population 3) Time horizon 4) Intervention
5) Comparator(s) 6) Scope of costs 7) Assumptions 8) Sensitivity analysis 9) Discounting 10) Validation
11) Data sources

1. ***Exclusion criteria***

Studies are excluded if they;

- Describe a review, background or a theoretical study about budget impact analyses.
- Report a health technology analysis other than a budget impact i.e. health technology assessment, cost-effectiveness analysis, cost study or value assessment framework.
- Are unrelated to budget impact analysis.
- Describe an intervention other than a drug.
- Report a budget impact analysis of a drug with no orphan status in the USA or EU.
- Combine orphan and non-orphan drugs in their budget impact analysis.
- Were withdrawn.

1. ***Risk of bias (quality) assessment***

This systematic review will be based on ISPOR good practice guidelines, a widely adopted standard for budget impact analyses. The quality assessment will be evaluated by a second reviewer and uncertainties are resolved
through discussion.

**Search strategy**

| **Connector** | **Field** | **Search Terms** | **Items** |
| --- | --- | --- | --- |
|  | All fields | (("budgets"[Mesh] OR budget*[tiab])) | 270 |
| AND | All fields | ("Rare Diseases"[Mesh] OR rare-disease*[tiab] OR rare[tiab] OR orphan-disease*[tiab] OR orphan[tiab] OR "Orphan Drug Production"[Mesh]) |  |

PubMed (searched on 16^th^ of January 2020)

| **History** | **Search** | **Results** |
| --- | --- | --- |
| #1 | ‘budget’/exp OR ‘budget*’:ti,ab,kw | 702 |
| #2 | 'rare disease'/exp OR ‘rare disease*’:ti,ab,kw OR ‘rare’:ti,ab,kw OR ‘orphan disease*’:ti,ab,kw OR ‘orphan’:ti,ab,kw OR 'orphan drug'/exp |  |
| #3 | #1 AND #2 |  |

Embase (searched on 16^th^ of January 2020)

| **Connector** | **Search Terms** | **Content** | **Results** |
| --- | --- | --- | --- |
|  | budget | All content | 979 |
| AND | rare disease OR rare OR orphan disease OR orphan OR orphan drug | All content |  |

Value in Health, ISPOR conference abstracts (searched on 16^th^ of January 2020)
